# Resequencing of Common Bean Identifies Regions of Inter–Gene Pool Introgression and Provides Comprehensive Resources for Molecular Breeding

**DOI:** 10.3835/plantgenome2017.08.0068

**Published:** 2018-07-01

**Authors:** Juan David Lobaton, Tamara Miller, Juanita Gil, Daniel Ariza, Juan Fernando de la Hoz, Alvaro Soler, Steve Beebe, Jorge Duitama, Paul Gepts, Bodo Raatz

**Affiliations:** ^1^ Agrobiodiversity Research Area, CIAT Cali 763537 Colombia; ^2^ Dep. of Plant Sciences Univ. of California Davis 95616 CA; ^3^ Systems and Computing Engineering Dep. Univ. de los Andes Bogotá 111711003 Colombia

## Abstract

Common bean (*Phaseolus vulgaris* L.) is the most important grain legume for human consumption and is a major nutrition source in the tropics. Because bean production is reduced by both abiotic and biotic constraints, current breeding efforts are focused on the development of improved varieties with tolerance to these stresses. We characterized materials from different breeding programs spanning three continents to understand their sequence diversity and advance the development of molecular breeding tools. For this, 37 varieties belonging to *P. vulgaris*, *Phaseolus acutifolius* (A. Gray), and *Phaseolus coccineus* L. were sequenced by whole‐genome sequencing, identifying more than 40 million genomic variants. Evaluation of nuclear DNA content and analysis of copy number variation revealed important differences in genomic content not only between *P. vulgaris* and the two other domesticated *Phaseolus* species, but also within *P. vulgaris*, affecting hundreds of protein‐coding genomic regions. A large number of inter–gene pool introgressions were identified. Furthermore, interspecific introgressions for disease resistance in breeding lines were mapped. Evaluation of newly developed single nucleotide polymorphism markers within previously discovered quantitative trait loci for common bacterial blight and angular leaf spot provides improved specificity to tag sources of resistance to these diseases. We expect that this dataset will provide a deeper molecular understanding of breeding germplasm and deliver molecular tools for germplasm development, aiming to increase the efficiency of bean breeding programs.

**Abbreviations**
ALS: angular leaf spot
BCMV: *Bean common mosaic virus*
CBB: common bacterial blight
CNV: copy number variant
HRM: high resolution melt
KASP: Kompetitive allele‐specific polymerase chain reaction
MAS: marker‐assisted selection
NABE: Namulonge Bean
NGSEP: Next Generation Sequencing Experience Platform
PCR: polymerase chain reaction
QTL: quantitative trait loci
RD: read depth
RIL: recombinant inbred line
SCAR: sequence‐characterized amplified region
SNP: single nucleotide polymorphism
UC: University of California
VAX: Vulgaris Acutifolious Xantomonas
WGS: whole‐genome (re)sequencing

## Core Ideas

- Inter–gene pool introgressions illustrate multiple admixture events and the breeding history of beans.
- A catalog of 40 million genomic variants is available for research on bean genetics.
- Genome‐wide variability is a valuable tool for molecular marker development.

Common bean is the most important grain legume for direct human consumption and is a staple food in different countries in Latin America and Eastern and Southern Africa (Broughton et al., 2003). Because common bean (like other grain legumes) is a major source of protein and micronutrients complementing the caloric contribution of cereal and root crops, this crop has a particular importance for resource‐limited smallholder farmers living in tropical regions that are characterized by widespread poverty and malnutrition. For this reason, several breeding programs have continually attempted to develop improved germplasm. The main trait is improved yields, which are currently limited by different biotic and abiotic stresses, as well as improving the nutritional value and grain quality of common bean (Beebe, 2012).

The use of genetic tools for breeding holds the potential to accelerate and reduce the costs of developing improved varieties for common bean as well as for other crops. A large number of genetic studies investigating diversity and quantitative trait loci (QTL) for different agronomic traits have been published in common bean (Beebe, 2012), resulting in a plethora of candidate markers to aid breeding by marker‐assisted selection (MAS). Genetic markers related to single‐gene traits have been successfully used in MAS, mainly for resistance to diseases (Singh & Schwartz, 2010). The progressive reduction of sequencing costs over the last decade has allowed us nowadays to carry out whole‐genome (re)sequencing (WGS) efforts, thus providing a deep understanding of the structure of nearly complete genomes across populations. Common bean was the first species in which more than one domestication was unequivocally demonstrated (Gepts et al., 1986) and multiple studies subsequently). Later studies demonstrated that the two domestications (in Mesoamerica and the southern Andes) took place from already diverged wild ancestors (Gepts, 1998; Bitocchi et al., 2012), both originating from a potentially extinct common ancestor in Mesoamerica (Ariani et al., 2018). A major breakthrough in common bean genetics were the recently published reference sequences of the Andean landrace ‘G19833’ (Schmutz et al., 2014) and the Mesoamerican breeding line ‘BAT 93’ (Vlasova et al., 2016).

Large WGS projects have been recently conducted on different crops such as rice (*Oryza sativa* L.) (Huang et al., 2012; Duitama et al., 2015), sorghum [*Sorghum bicolor* (L.) Moench.] (Mace et al., 2013), bell pepper (*Capsicum annuum* L.)(Qin et al., 2014), tomato (*Solanum lycopersicum* L.) (Lin et al., 2014), and even in other legumes such as lupin (*Lupinus angustifolius* L.) (Yang et al., 2015). These studies revealed the footprints of domestication and population dynamics within each species and allowed the assembly of large‐scale databases of intra‐species genomic variation including millions of single nucleotide polymorphisms (SNPs) and thousands of small indels, facilitating, in some cases, the genetic mapping of agronomically relevant traits through genome‐wide association studies. For breeding purposes, WGS of elite cultivars is a promising strategy to facilitate the design of genetic markers for MAS of loci with large additive effects on the traits of interest (Duitama et al., 2015; Yang et al., 2015). Especially for autogamous species with relatively small genomes such as rice or common bean, WGS achieves an almost complete reconstruction of genic regions, allowing the correlation of phenotypic variation with haplotypes representing beneficial alleles for the trait of interest. This facilitates the design of SNP markers, either within or very close to causative sequences, which can be used to track and accumulate beneficial alleles through MAS.

Current breeding efforts in *P. vulgaris* include introgression of desirable characteristics of the species *P. coccineus* and *P. acutifolius* from the secondary and tertiary gene pools, respectively. These hold a reservoir of valuable alleles for the improvement of common bean (Beebe, 2012). Breeding lines developed from crosses with *P. coccineus* achieved high aluminum tolerance and superior root growth (Butare et al., 2011). Likewise, breeding lines developed from interspecific crosses with *P. acutifolius* have been used to introgress resistance to common bacterial blight (CBB) and are promising for drought tolerance (Singh et al., 2001). Common bacterial blight caused by *Xanthomonas campestris* pv. *phaseoli Smith* (Dye) and *Xanthomonas fuscans subsp. fuscans* sp., is a severe disease of major agronomic and economic importance for common bean (Duncan et al., 2012). Because genetic resistance to CBB is relatively low in *P. vulgaris* compared with *P. acutifolius*, breeding lines with acceptable levels of resistance have been developed from crosses with *P. acutifolius* (Miklas et al., 2011; Viteri et al., 2015).

Angular leaf spot (ALS) is another economically relevant disease for common bean production, caused by the fungus *Pseudocercospora griseola*. Yield losses of up to 80% have been reported as a result of ALS infection across bean‐producing areas of the world (Sartorato et al., 2000; Lemessa et al., 2011). Unlike the case for CBB, resistance to different pathotypes of *P. griseola* has been identified in several common bean genotypes. Single dominant resistance loci have been reported as well as more quantitative resistance controlled by several loci (Mahuku et al., 2004, 2009; Gonçalves‐Vidigal et al., 2011; Ddamulira et al., 2014; Keller et al., 2015). Sources of resistance to ALS include the Guatemalan accession ‘G10474’, which is resistant to isolates from different locations, including the highly pathogenic race 63–63 (Mahuku et al., 2004). A single dominant gene on chromosome Pv08, tagged by the sequence‐characterized amplified region (SCAR) marker PF5 at a distance of 5 cM has been reported as tagging a major ALS resistance gene (Mahuku et al., 2004). Gonçalves‐Vidigal et al. (2011) illustrated the capability of a marker database like PhaseolusGenes (http://phaseolusgenes.bioinformatics.ucdavis.edu, accessed 22 Feb. 2018) to speed up marker identification. In the Andean gene pool, important resistance loci have been identified in the accession ‘G5686’, showing resistance against several Andean and Mesoamerican isolates of *P. griseola* (Mahuku et al., 2009). Single nucleotide polymorphism markers tagging loci from G5686 were recently developed (Keller et al., 2015). Ddamulira et al. (2014) observed high levels of ALS pathogen variability and resistance among indigenous landraces in Uganda, setting the stage for the discovery of additional resistances and the underlying genes.

This study aims at the genetic characterization of breeding material from different breeding programs spanning several continents to understand sequence diversity and advance the development of molecular breeding tools by using the unprecedented depths of sequencing information. In a joint effort by several institutes, we performed WGS and bioinformatic analysis on 35 *P. vulgaris* varieties, landraces, and elite breeding lines, as well as two genotypes of *P. acutifolius* and *P. coccineus*. Important sources of resistance to pathogens and abiotic stress tolerance, as well as highly performing varieties, were included in this work. Including available data from previous studies, we identified more than 40 million sites with evidence of genomic variation within the sequenced samples. To illustrate the application of genomic tools in bean breeding, we analyzed several features of the bean genome, including genetic diversity, total genome sequence size, inter–gene pool introgressions, and copy number variation (CNV). We also provide examples of breeding tool development that were based on this genome sequence information.

## Materials and Methods

### Germplasm

Germplasm was selected for sequencing on the basis of agronomic importance, being parents of genetic or breeding populations, and harboring valuable traits for biotic and abiotic stress resistance. Twenty genotypes were selected and extracted at University of California (UC) Davis, Davis, CA, USA, and 17 at CIAT, Colombia. Supplemental Table S1 lists detailed information about characteristics of each line.

### DNA Extraction and Sequencing

For CIAT samples, total genomic DNA was extracted from 1 g of leaf tissue of 30‐d‐old plants using liquid N and the urea buffer‐based DNA extraction midi prep protocol (Chen et al., 1992). DNA was quantified with NanoDrop 2000 (Thermo Fisher Scientific, Waltham, MA) and the DNA quality was evaluated by electrophoresis and visualization on a 1% agarose gel, including digestions with *Eco*RI, PstI, and RsaI enzymes. Sequencing libraries for eight accessions were prepared with the TruSeq DNA PCR‐Free Library preparation kit (Illumina, San Diego, CA) and sequencing was performed at the HudsonAlpha Institute for Biotechnology (hudsonalpha.org/, accessed 22 Feb. 2018). For the other nine accessions, WGS was performed on the HISeq 2000 system (Illumina) by the Yale Center for Genome Analysis (http://medicine.yale.edu/keck/ycga/index.aspx, accessed 22 Feb. 2018). For UC Davis samples, DNA was extracted from freeze‐dried bean leaves of greenhouse‐grown plants using the Qiagen DNeasy Plant Mini Kit (#69106 Qiagen, Hilden, Germany) and DNA quality was assessed by 1% agarose gel electrophoresis. DNA with an absorbance ratio (A_260_:A_280_) of >1.7 and with no visible degradation on the agarose gel was used for subsequent library preparation. Libraries were prepared and sequenced at the QB3 Vincent J. Coates Genomic Sequencing Laboratory at UC Berkeley (Berkeley, CA; http://qb3.berkeley.edu/gsl/, accessed 22 Feb. 2018). Libraries were prepared from purified genomic DNA with the TruSeq DNA PCR‐Free Library preparation kit Illumina, San Diego, CA) and quantified with the QUBIT dsDNA HS assay kit (Thermo Fisher Scientific, Portsmouth, NH). Libraries were sequenced on an Illumina HiSeq 2500, with 300‐bp paired end fragment length.

### Catalog of Repetitive Regions for Filtering of Genomic Variants

We used two complementary sources to identify regions that can be considered as repetitive in the reference genome. First, we extracted the genomic locations of lowercase sequences in the “softmasked” version of the reference genome version 2.1 available in Phytozome (Phytozome, 2017). We found a total of 324,854 regions spanning 257 Mbp (47.9%) of the current 537‐Mbp assembly. Alternatively, we aligned the Illumina reads from G19833 that had previously been generated to build the initial version of the reference genome (Schmutz et al., 2014) to the new reference genome and we ran the Next Generation Sequencing Experience Platform (NGSEP) command FindVariants (Duitama et al., 2014), using the options “‐noRD” “‐noRP”, and “‐noSNVS”. In this mode, FindVariants activates an algorithm that clusters overlapping nonunique alignments and reports the initial and final coordinate of each cluster as a nonunique region for alignment purposes. This procedure produced 36,340 regions spanning 255 Mbp (47.5%) of the bean genome. The intersection between these datasets was 204 Mbp, which corresponds to 79% of each dataset. Following a conservative approach to determine genomic variants in nonrepetitive regions of the genome, we described any region within the union of the two sets of repeats described above as repetitive. After merging redundant and overlapping segments, the merged set of repeats included 254,106 regions spanning 309 Mbp (57.5%) of the bean genome. The three sets of repeats are available in dryad (10.5061/dryad.46pk7, accessed 6 Mar. 2018) (see the “Data Availability” section for details) for custom variant filtering.

### Read Alignment and Variant Detection and Genotyping

The recently released version 2.1 of the G19833 Andean reference genome of *P. vulgaris* (Schmutz et al., 2014) was downloaded from the phytozome website (Phytozome, 2017). Raw reads obtained from the sequencing process were aligned to the reference genome with bowtie2 version 2.3.2 (Langmead & Salzberg, 2012). Most of the parameters of bowtie2 were left with their default values, with the exception of the maximum number of alignments per read, which was set to 3 (Supplemental Script S1), and the minimum and maximum fragment lengths, which was set independently for each sample (Supplemental Table S2). For the samples G35346 and G40001 of the related species *P. coccineus* and *P. acutifolius*, alternative parameters similar to those suggested in the “very sensitive” mode described in the bowtie2 manual were tried (Supplemental Script S2).

To discover and genotype SNPs, small indels, and CNVs the NGSEP pipeline version 3.0.2 (Duitama et al., 2014) was executed. The NGSEP pipeline discovers genetic variants and predicts the genotype (either homozygous for one allele or heterozygous) of each sample within each genetic variant (SNP, small indel, or short tandem repeat), based on the evidence provided by the aligned base pairs spanning such loci and the corresponding base quality scores of these base pairs. This procedure yields a matrix of predictions, termed genotype calls, which have as many rows as variant loci and as many columns as sequenced samples. Each entry in this matrix is called with a certain confidence, termed genotype quality, which is usually reported as a Phred score. Genotype calls with quality lower than a minimum desired threshold are considered to be missing data [see Duitama et al. (2014) for more details]. The NGSEP was executed with its recommended parameters for analysis of WGS data: (i) minimum genotype quality: 40; (ii) maximum value allowed for a base quality score: 30; and (iii) a maximum number alignments allowed to start at the same reference site: 2. To improve the accuracy of the variant discovery step, a catalog of predicted short tandem repeats was assembled with Tandem Repeat Finder version 409 software (Benson, 1999) with the parameters recommended in the current version. Predicted short tandem repeats with unit lengths larger than 3 and less than three copies or a low score (less than 40) were filtered out (Supplemental Script S3). The rationale for this filter is that the tandem repeats with short unit lengths are more likely to produce misalignments that generate false positive variants. For individual samples (excluding pools), the prior heterozygosity rate (‐h option) was set to 0.0001 to give a larger prior probability to homozygous genotypes (Supplemental Script S4 and Supplemental Script S5). For pooled samples, the prior heterozygosity of 0.001 was left by default (Supplemental Script S6). The number of base pairs to ignore in the 5’ end and in the 3’ end of the reads was set independently for each sample (Supplemental Table S2) based on the distribution of the differences from the reference per sequencing cycle provided by the QualStats command of NGSEP (Supplemental Fig. S1).

The final catalog of SNPs, small indels, and short tandem repeats was generated by running the command MergeVariants in NGSEP, using the VCF files generated in the discovery step as input (Supplemental Script S4, Supplemental Script S5, and Supplemental Script S6). The variants were then genotyped on each sample, again with the command FindVariants of NGSEP providing the catalog of known variants and the same parameters used in the discovery step, except for the minimum quality, which was set to zero to retain the maximum amount of information on the genotype calls and to try different quality options at the filtering step (Supplemental Script S7, Supplemental Script S8, and Supplemental Script S9). After individual genotyping, the final variation dataset was assembled with the MergeVCF command of NGSEP.

The NGSEP commands Annotate, FilterVCF, and ConvertVCF were used, respectively, to perform functional annotation of variants, to apply the different filters described for the different downstream analyses, and to convert VCF files to other formats. The command IntrogressionAnalysis of NGSEP was used to identify SNPs in which the difference in the reference allele frequency between Andean and Mesoamerican samples was larger than 0.9 (option‐d 0.9 and‐v were used to print a VCF with the discriminative SNPs). A file with the background population of each accession was provided to this command to derive the reference and alternative allele frequencies within each population. See more details about this module in the next section. To build distance‐based unrooted dendrograms, the command ConvertVCF of NGSEP was used to convert the VCF file to a fasta file. The tool PGDSpider (Lischer and Excoffier, 2012) was then used to convert the fasta file to nexus format. The neighbor‐joining algorithm implemented in SplitsTree4 (Huson & Bryant, 2006) was then used to build the dendrogram. Finally, Flapjack (Milne et al., 2010) was used for visualization of the variation dataset at specific sites of the genome.

### Introgressions

For introgression analysis within *P. vulgaris*, we first selected the SNPs in nonrepetitive regions of the genome, and genotyped in at least 50 *P. vulgaris* accessions by running the FilterVCF command of NGSEP using the options “‐frs” and “‐minI”. Single nucleotide polymorphisms within regions with predicted CNVs based on the read depth (RD) analysis were also removed with the option “‐maxCNVs 0”. To remove low‐frequency variants, increasing the number of possible haplotypes, we removed the SNPs with minor allele frequency below 0.1 (option “‐minMAF 0.1)”. We then selected SNPs for which a Mesoamerican and an Andean allele could be clearly identified and hence the haplotypes composed of such SNPs could be clearly assigned to either the Mesoamerican or the Andean population. Because the individual accessions, particularly the breeding lines, were expected to hold foreign haplotypes in certain regions of the genome, instead of comparing allele frequencies on the individual accessions, we selected 63,247 SNPs in which the NGSEP genotype calls for the wild Andean and the wild Mesoamerican pool were contrasting and homozygous. The rationale for following this procedure is that the accessions sequenced within the wild pools were not expected to hold introgressions and that a SNP for which NGSEP provided a homozygous genotype call within a pool was expected to be conserved in the population sequenced within such pool. The density of the selected SNPs in 1‐Mbp windows across the genome is shown in Supplemental Fig. S6. Finally, we ran the module for introgression analysis available in NGSEP version 3.0.2 (command IntrogressionAnalysis) to predict the genomic regions with introgressions, providing a VCF file with the SNPs selected above and a tab‐delimited text file describing the background population of each accession (Supplemental Table S1). This module divides the genome into nonoverlapping windows of 50 SNPs (by default). For each window, it implemented the following heuristic approach. It calculated the characteristic haplotype of each population by calculating the major allele of each SNP within this population. For each sample, it then calculated the similarity score of the sample haplotype against the representative haplotype of each population with a default a score of +1 for one match, ‐1 for one mismatch, and zero for missing data. The sample haplotype was assigned to the population with maximum score if this score passed a minimum threshold (30 by default) and also if the difference with the second score was larger than another threshold (10 by default). Whereas haplotypes not passing the former condition were cataloged as unassigned, haplotypes not passing the latter condition are cataloged as undecided. Introgressions were called for each sample on a series of windows (at least one) for which the assigned population of the sample haplotype was different from the background population provided.

### Genome Size Analysis

Genome sizes were determined with a PARTEC flow cytometer (Sysmex Partec GmbH, Görlitz, Germany). We determined the relative genome size of the reference genome *P. vulgaris* G19833 and two sister *Phaseolus* species by parallel analysis. Rice (*Oryza sativa* cv. Nipponbare) was included as a known size reference (395 Mbp) for calibration of the equipment (Kawahara et al., 2013).

For nucleus extraction and DNA staining, 0.5 cm^2^ of young and healthy leaf tissue was chopped in 500 μL of an extraction buffer according to the manufacturer's protocol (cyStain Propidium Iodide reagent kit; Sysmex Partec GmbH). Samples were filtered through a 50‐μm nylon filter and incubated in the dark for at least 30 min in 2 mL of staining solution. Nuclear DNA was quantified in comparison to the reference G19833, averaging three technical replicates from three biological replicates evaluated on three consecutive days.

### Analysis of CNV

Copy number variations were identified by running the RD analysis available in NGSEP with default parameters for 37 accessions representing individual varieties that clearly clustered within the Andean or the Mesoamerican populations and also removing the 18 accessions sequenced at low average RD by Song et al. (2015). After inspecting the overlap between the regions with CNV predictions for each sample and the nonrepetitive regions of the genome, the samples ‘Midas’ and ‘SXB 412’ were excluded from the rest of the analysis (Supplemental Fig. S3). Per‐sample CNV predictions were merged via a heuristic clustering method implemented in a custom script. In brief, two CNV calls in two different varieties were considered as potentially representing the same event if the intersection between their affected regions was ≥70% of the length of each region. This defined an undirected consistency graph for each set of overlapping calls. A greedy algorithm was then implemented to extract cliques of large numbers of nodes progressively. At each step, the graph of unclustered nodes was built and the nodes were sorted and ordered by degree from largest to smallest. The degree of each node limited the length of the clique that could be formed with this node as a pivot. Cliques were identified and compared for each node until the degree of the node was smaller than the maximum clique found up to that point. Each clique represented a single predicted CNV. Once the clusters of at least two calls had been identified, the remaining unclustered CNV calls were added to the cluster with the largest percentage of consistent calls if this percentage was larger than 50%. Otherwise, the call was treated as unique for its corresponding variety and a separate cluster was formed for such CNV call. This procedure ensured that every call was taken into account in the final dataset. Allele dosages for the CNVs identified via this merging procedure were calculated for each variety, again running the FindVariants command of NGSEP with the option “‐knownSVs” providing the catalog of merged CNVs used as input. To estimate a quality score for each call, conditional probabilities for zero to nine alleles were calculated as the *p*‐values of a *t*‐test on the predicted allele dosage (as a real number) with the assumed number of alleles as the mean and the same SD as estimated from the distribution of the RD signal over the genome. Under the assumption of a uniform prior, the Phred score of the largest posterior probability was used as quality score for each genotype call.

This procedure led to a matrix of predictions of copy number, which we term the CNV genotype calls. These had as many rows as CNVs and as many columns as analyzed samples. Three subsequent filters were applied to try to select high‐quality CNVs on the basis of the background genomic region and the consistency of the predictions of copy number across the 35 accessions: (i) Keep CNVs in which less than 30% of the region spanned repetitive elements; (ii) keep CNVs that have at least 30 samples genotyped; and (iii) keep only CNVs with at most two values of copy number (biallelic) over the genotyped varieties and that have a predicted copy number different from 2 in at least one variety. Regarding the third filter, although, in principle, there should not be differences in quality between biallelic and multiallelic CNVs, for this particular dataset, it is reassuring to observe a consistent prediction of an alternative copy number (≠ 2) across different accessions sequenced in different centers at different times and when different DNA extraction and library preparation protocols were applied.

### Sequence‐Characterized Amplified Region Genotyping

A summary of all markers genotyped in this study is available in Supplemental Table S5. The SCAR marker PF5 was amplified by polymerase chain reaction (PCR) in 15‐μL reaction volumes containing 5 μL of genomic DNA solution (∼50 ng), 1× PCR buffer [10 mM tris(hydroxymethyl)‐aminomethane–HCl (pH 8.8), 50 mM KCl, 0.8% (v/v) Nonidet P40 (ThermoFisher Scientific), 2.5 mM of MgCl_2_ (ThermoFisher Scientific)], 0.4 mM of dNTPs mix (Promega, Madison, WI), 0.2 μM of each primer (forward and reverse), and 0.15 μL of Taq polymerase (lab made). The PCR was performed on an Eppendorf Pro or ProS Mastercycler (Eppendorf North America, Hauppauge, NY) under the following conditions: an initial denaturation step at 94°C for 5 min, followed by 35 cycles of denaturation at 94°C for 30 s, annealing at 60°C for 30 s, extension at 72°C for 45 s, and a final extension step at 72°C for 10 min. The SCAR markers SU91 and SAP6 were amplified by PCR on PTC‐200 thermocyclers (MJ‐Research, Bio‐Rad, Hercules, CA) in 20‐μL reaction volumes with 5 μL of the DNA solution (∼50 ng), 0.16 μM of each primer, 1.5 mM MgCl_2_, 0.6 mM dNTPs mix (Promega), and 1 U of Taq polymerase (Fermentas) in 1× PCR buffer [10 mM tris(hydroxymethyl)‐aminomethane–HCl (pH 8.8), 50 mM KCl, 0.1% TritonX‐100]. The thermocycling profile was 1 cycle of 2 min at 94°C, 30 cycles of 10 s at 92°C, 10 s at 50 to 60°C, and 25 s at 72°C, followed by one cycle of 5 min at 72°C. Polymerase chain reaction products were visualized on 1.5% agarose gels in 1× Tris‐Borate‐EDTA buffer on a HORIZON 20·25 gel electrophoresis system. GelRed (Biotium, Fremont, CA) was added to the gel for visualization of the DNA bands on an ultraviolet transilluminator (Foto/UV 26, Fotodyne Inc., Hartland, WI).

### Single Nucleotide Polymorphism Genotyping by Melting Temperature Shift Analysis

Primers were designed according to (Wang et al., 2005) using the software Primer 3 (Untergasser et al., 2012). The markers were amplified by PCR on a fluorescence‐detecting thermocycler (CFX384 Real‐Time System, Bio‐Rad) with EvaGreen fluorescent dye (Biotium). Melting point analysis for allele determination of the template DNA was performed with the same equipment. The PCR reaction volume was 15 μL containing 5 μL of genomic DNA, 1× Taq buffer, 2 mM of MgCl_2_, 0.2 mM of dNTPs mix (Promega), 0.2 μM of each primer (two allele‐specific forward primers and the common reverse primer), 0.6× EvaGreen (Biotium), and 0.1 μL of Taq polymerase (lab made) under the following thermal profile: an initial denaturation step at 94°C for 3 min, then 35 cycles of denaturation at 92°C for 20 s, annealing for 20 s (the temperature was specific to each primer trio), and extension at 72°C for 20 s, followed by 1 min at 95°C and the melting curve step ramping from 70 to 95°C in increments of 0.5°C every 20 s under continuous fluorescence detection.

### Single Nucleotide Polymorphism Genotyping by High‐Resolution Melting Analysis

High‐resolution melting (HRM) primers were designed to flank a SNP with an amplicon size not exceeding 100 bp. Polymerase chain reaction amplification was performed as described before for melting temperature shift genotyping on the CFX384 Real‐Time System (Bio‐Rad) with the difference that the amplification product was melted by ramping from 65 to 95°C in increments of 0.2°C every 20 s. High‐resolution melting analysis was performed with Bio‐Rad Precision Melt Analysis version 1.2 software (Wittwer et al., 2003).

### Single Nucleotide Polymorphism Genotyping with Kompetitive Allele‐Specific PCR Markers

Kompetitive allele‐specific PCR (KASP) chemistry (LGC Genomics, Hoddeson, UK) is based on using three primers (two allele‐specific and a common reverse primer), similar to melting temperature shift analysis, whereas different tags were used to incorporate the fluorescence‐labeled oligos. The KASP assays can be purchased from the commercial provider, LGC Genomics (http://www.lgcgroup.com/products/kasp‐genotyping‐chemistry, accessed 22 Feb. 2018) and run on real‐time PCR machines. For this project assay, the design and genotyping were performed by LGC Genomics.

### Data Availability

The raw data sequenced in this study are available at the NCBI sequence read archive database (http://www.ncbi.nlm.nih.gov/sra, accessed 22 Feb. 2018) with the bioproject accession numbers PRJNA294602 and PRJNA354243. The variants identified and genotyped in the samples sequenced in this study are available for downloading at dryad (10.5061/dryad.46pk7, accessed 6 Mar. 2018) and for browsing at the European Variation Archive database of the European Bioinformatics Institute with the accession number PRJEB18671.

## Results

### Whole‐Genome Resequencing

**Fig. 1:**
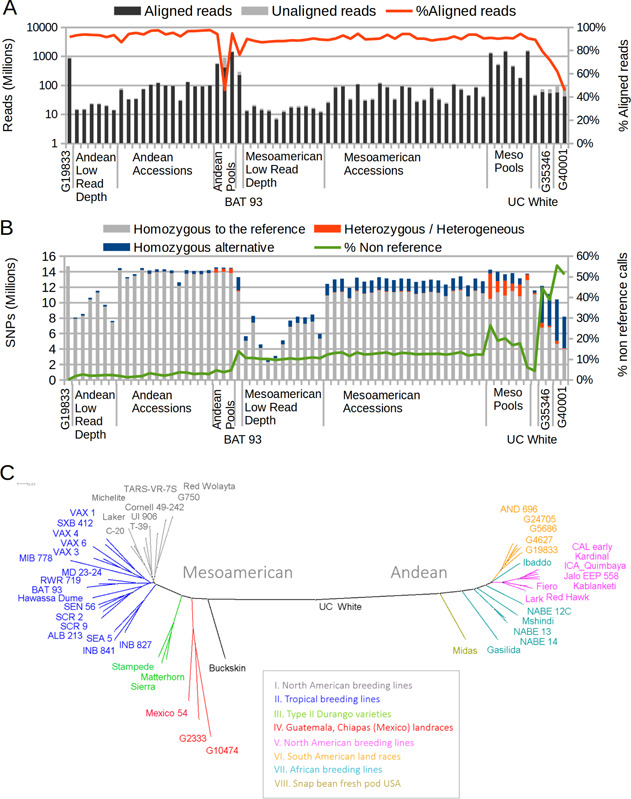
Variants discovery and diversity analysis. (A) Raw number of whole‐genome sequencing (WGS) reads analyzed for each sample and the percentage of reads aligned to the reference genome (red line). The number of aligned reads corresponds to the bold segment of each bar. Reads from samples G35346 and G40001 were subjected to default and more sensitive alignment parameters in bowtie2 to increase the number of reads aligned to the reference. (B) Number of single nucleotide polymorphisms (SNPs) that could be reliably genotyped in each sample from a dataset of SNPs in nonrepetitive regions of the genome. Genotype calls are discriminated as homozygous for an alternative (nonreference) allele (blue), heterozygous or heterogeneous (red), and homozygous for the reference allele (gray). The percentage of nonreference genotype calls is displayed as a green line. (C) Neighbor‐joining dendrogram of distances between the samples analyzed in this study based on genome‐wide high‐quality SNP markers.

We performed WGS on 37 *Phaseolus* accessions, including 22 *P. vulgaris* lines from the Mesoamerican gene pool and 13 from the Andean gene pool, and one accession each of the species *P. acutifolius* and *P. coccineus*, (Supplemental Table S1). Lines were selected on the basis of agronomic importance, being parental lines of breeding populations in Eastern Africa as part of the African Bean Consortium project, and resistance to diseases [ALS, CBB, *Bean common mosaic virus* (BCMV), rust, and anthracnose) and abiotic stresses (drought, heat, and low soil fertility). As alleles from the species *P. coccineus* and *P. acutifolius* were successfully introgressed in some *P. vulgaris* elite cultivars, WGS was also performed on one genotype of each of these species. Additionally, we reanalyzed the raw Illumina WGS data from three recently published studies: (i) Discovery of SNPs for genotyping using data from 18 *P. vulgaris* varieties sequenced at 2× to 5× average RD (Song et al., 2015); (ii) development of the *P. vulgaris* reference genome, including pooled resequencing of 100 landraces and 60 wild relatives distributed in eight pools to assess genomic variability within bean subpopulations (Schmutz et al., 2014); and (iii) de novo assembly of the Mesoamerican breeding line BAT 93 (Vlasova et al., 2016). The complete raw data included approximately 13 billion paired‐end Illumina reads with lengths between 76 and 152 bp and an average RD per sample between 1.63× and 311× (Fig. 1). These reads were aligned to the recently released *P. vulgaris* reference genome version 2.1 available from Phytozome (2017) with bowtie2 (Langmead & Salzberg, 2012). Except for the South Andean pool and the Mesoamerican genotype BAT 93, the alignment rates for every *P. vulgaris* sample were above 85% (Fig. 1A, Supplemental Table S2). Excluding these outliers, the alignment rates for Andean samples (94% on average) were somewhat higher than those obtained for the Mesoamerican samples (91% on average), which is expected, as the reference genome corresponds to a genome assembly of the Andean accession G19833. The alignment rate for G19833 reads was 92%. About half of the unaligned reads could be aligned to a chloroplast sequence of *P. vulgaris* and a mitochondrial sequence of *Vigna radiata* (L.) R. Wilczek, available in the NCBI nucleotide database. This suggests that unaligned reads were mostly sequenced from DNA of organelles, which are not represented in the current reference sequence. With the default parameters for bowtie2, the alignment rates for *P. coccineus* and *P. acutifolius* were 72 and 45% respectively, consistent with their respective phylogenetic distances from *P. vulgaris* (Delgado‐Salinas et al., 2006). By testing different options to increase the sensitivity of bowtie2, we increased the alignment rates to 79 and 62% for *P. coccineus* and *P. acutifolius,* respectively, at the potential cost of increasing false positive variants. Reads that did not align to the bean reference genome, even after using sensitive parameters, are likely to be genome regions of *P. coccineus* and *P. acutifolius* that are not present in *P. vulgaris*. Alignment rates to the initial version 1.0 of the reference genome (Schmutz et al., 2014) were about 2% lower for all samples but preserved the same general pattern of variation. Sequencing quality plots for each sample showed that the average percentage of base calls that were different from the reference sequence was close to 0.5% for all the Andean genotypes (including G19833), about 1.2% for the Mesoamerican genotypes, and 2.7 and 3.5% for *P. coccineus* and *P. acutifolius,* respectively (Supplemental Fig. S1). The agreement between the genetic background of the samples and the average number of differences from the reference, regardless of the group in which each sample was sequenced, indicates that these differences can mainly be explained by real variation against the reference and not by sequencing errors. Consistent with other studies, the error rates tended to increase toward the 3’ end of the reads.

### Variant Detection

By using the NGSEP pipeline (Duitama et al., 2014), we assembled a dataset of sequence variation across the genomes of our plant samples. Details of the information contained on datasets of genomic variation and on the procedure followed to build the dataset presented in this work can be found in the Materials and Methods section. The assembled dataset included 45 million biallelic SNPs, 2.1 million biallelic indels, and 3.7 million multiallelic variants. If we considered only the nonrepetitive regions of the genome, the number of variants decreased to 14.7 million biallelic SNPs, 1.3 million biallelic indels, and 1.4 million multiallelic variants. Figure 1B shows the number of genotype calls that could be obtained confidently for each sample within the biallelic SNP markers in nonrepetitive regions of the genome (see the exact numbers per accession in Supplemental Table S2). The calls are discriminated by the three possible genotypes for a biallelic site in a diploid individual: (i) Homozygous and identical to the reference allele, (ii) heterozygous, and (iii) homozygous but different from the reference. The total number of genotype calls per sample is correlated with the raw number of reads. Whereas more than 12 million SNPs (>81%) could be genotyped for samples with more than 25 million raw reads (around 5× average RD), the number of SNPs genotyped declined as the number of raw reads decreased. By removing the accessions with low average RD and removing the pools, on average 95% of the SNPs in this dataset could be genotyped for Andean varieties, whereas this percentage was 87% for Mesoamerican varieties, which is consistent with the known phylogenetic and population structure of *P. vulgaris* (Delgado‐Salinas et al., 2006; Kwak & Gepts, 2009; Mamidi et al., 2011). Moreover, the percentage of nonreference SNP genotype calls per accession was below 5% for all Andean samples (including the pools), but increased to over 10% for every Mesoamerican sample and up to 45 and 55% for *P. coccineus* and *P. acutifolius*, respectively. For individually sequenced *P. vulgaris* lines, which are mostly inbred, heterozygosity is expected to be rare; in the pools, the calls most probably reflected heterogeneity among the samples included in the pool rather than heterozygosity of the individual lines included in each pool. Expectedly, the percentages of heterogeneous genotype calls within the pools (3–23%) were larger than the percentages of heterozygous calls for individual *P. vulgaris* samples, which were, on average, 0.17% for Andean varieties and 0.46% for Mesoamerican varieties. If we removed the outgroup species, the number of SNPs within *P. vulgaris* decreased to 23 million overall and 1.3 million in nonrepetitive regions. Within this dataset, the percentage of nonreference genotype calls in Andean samples increased to 6.31% on average, whereas the percentage of nonreference calls for Mesoamerican lines increased to 32.32% on average (Supplemental Fig. S2). As expected, the largest percentage of both nonreference and heterogeneous or heterozygous calls was observed in the wild Mesoamerican pool (65 and 56% respectively). A total of 13,261 SNPs perfectly distinguished the Andean and Mesoamerican accessions.

We checked if the WGS variation dataset described here contained SNP markers previously developed for high‐throughput genotyping (Goretti et al., 2014; Song et al., 2015). The coordinates of these SNP markers were located relative to the previous version of the reference genome (version 1.0). Hence, we first realigned the primer sequences of each SNP to recalculate its genomic coordinates relative to the reference genome version 2.1. We then searched these predicted coordinates in the WGS variation dataset. The genomic coordinates of all 60 SNPs published by Goretti et al. (2014) could be identified and from these and 47 (78%) could be consistently recovered in the WGS dataset. Within the 6000 SNPs identified in Song et al. (2015), genomic coordinates were identified for 5991; 5638 (94%) appear in the WGS dataset. The published reference and alternative alleles of all SNP markers found in the WGS dataset are consistent with those predicted from the WGS data. Unfortunately, the previously released SNPs did not indicate individual genotyping per accession to allow us to perform comparisons at the level of genotype calls. Taking into account the potential loss of variants caused by the change in reference genome, the large percentage of recovery of the SNP markers released in previous works is a good indicator of the reliability of the WGS variation dataset assembled in this study.

### Genetic Relationships among Individual *P. vulgaris* Lines

By selecting only the dataset of SNPs within the individual 55 *P. vulgaris* accessions (removing the pools and the related species), we applied further filters by removing any variants genotyped in less than 50 individuals and any variants located less than 10 bp from another variant. We performed a clustering analysis based on this high‐quality genotype dataset and built a neighbor joining dendrogram (Fig. 1C). As expected, the dendrogram showed two major clusters, representing the Mesoamerican and Andean gene pools. In the Mesoamerican gene pool, two clusters of North American varieties could be observed: The Type II Durango race entries (Group III: ‘Sierra’, ‘Stampede’, and ‘Matterhorn’) and the Mesoamerican black‐ and white‐seeded varieties (Group I: black: ‘T‐39’, ‘Cornell 49–242’; white: ‘Michelite’, ‘C‐20’, ‘Laker’). Lines from the CIAT breeding program appear in a separate group (Group II). The genetically most distant are the lines from Guatemala or adjacent Chiapas (Mexico), which probably represent the race Guatemala (Beebe et al., 2000). The lines forming this cluster (Group IV: ‘G2333’, ‘G10474’, and ‘Mexico 54’), are important disease resistance donors for ALS and anthracnose.

It is interesting to note the position of some additional lines in this dendrogram. The Guatemalan accession ‘G750’, a rare Mesoamerican determinate accession with an unusual *PvTFL1y* haplotype (Kwak et al., 2012), appears to belong to the Mesoamerican race in Group I. Group I also contains ‘Red Wolayta’, whereas Group II contains ‘Hawassa Dume’, a variety from Ethiopia, and ‘RWR 719’, a landrace from Rwanda with resistance to *Pythium* root rot.

Andean accessions mainly split into four clusters. One included mainly North American varieties (Group V: e.g., ‘Cal Early’, ‘Kardinal’, ‘Red Hawk’, and ‘Lark’). It does, however, also include a variety derived from a Brazilian landrace (Jalo EEP558). A second group includes South American landraces (Group VI: ‘G4627’, G5686, G19833, and ‘G24705’). Although the North American landraces tended to form tight clusters, indicating low genetic variability, African varieties were mostly scattered over all groups. As detailed in the next section, the Andean Namulonge Bean (NABE) lines, the ‘Mshindi’, ‘Ibaddo’, and ‘Gasilida’ varieties (Group VII) departed from the previous two Andean clusters, potentially because of small‐sample effect and also local hybridization. The variety Mshindi is a BCMV‐resistant derivative of ‘Kablanketi’ (a Tanzanian landrace in Group V) obtained through crossing with ‘Rojo’, an inter–gene pool hybrid. Midas is a US snap bean cultivar cultivated for its yellow fresh pods (Group VIII).

### Evaluation of Inter–Gene Pool and Interspecific Introgressions

**Fig. 2:**
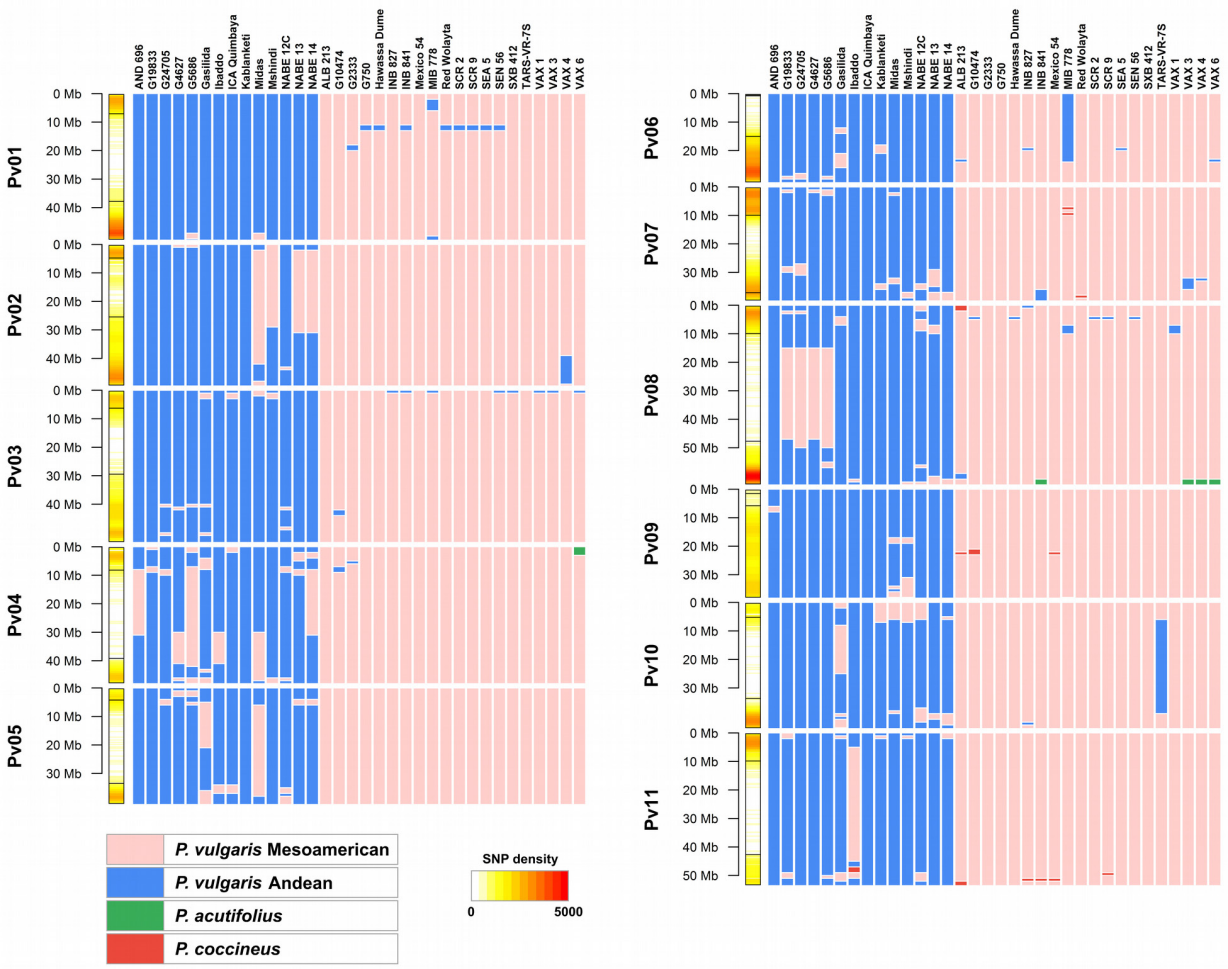
Inter–gene pool and interspecifc introgressions in common bean lines. Representation of inter–gene pool introgressions between 15 Andean genotypes and 23 Mesoamerican genotypes. The background Andean haplotype (blue), the background Mesoamerican haplotype (pink), and the regions of introgression of *P. acutifolius* (green) and *P. coccineus* (red) haplotypes are represented along the 11 chromosomes. The heat map at the left side of each chromosome represents the single nucleotide polymorphism (SNP) density for each region. Pericentromeric regions are shown in black boxes.

Some of the accessions sequenced in this study corresponded to improved materials developed by classical breeding techniques (i.e., cross‐hybridization and selection). For this reason, it is expected that the cultivars that have parents from both the Mesoamerican and Andean gene pools will keep large haplotype segments derived from these two populations. In addition, some of the accessions included in this study are landraces with an unknown pedigree (e.g., G19833, G5686, ‘G2595’, G2333, G4627, and G24705) but which may have undergone inter–gene pool hybridizations. To reveal these haplotype segments in the improved varieties or landraces, we performed a window‐based genome scan to identify foreign haplotypes in the accessions sequenced in this study (see the Materials and Methods for details). We identified 203 potential introgression events, 100 of which spanned over 1 Mbp of the genome and 27 spanned more than 5 Mbp (Fig. 2, Supplemental Table S3). Whereas 155 events corresponded to Mesoamerican introgressions in 14 varieties with an Andean background, 48 corresponded to Andean introgressions in 19 Mesoamerican varieties. The Andean cultivar Ibaddo contained the largest introgression, covering almost the entire chromosome Pv11. The Andean cultivar Midas shows the largest amount of Mesoamerican introgressions covering 95.7 Mbp of the genome. This explains the placement of Midas separate from the other Andean varieties in the dendrogram built from genome‐wide markers (Fig. 1C). Together with this genotype, the African varieties Mshindi, ‘NABE 13’, and ‘NABE 14’ share a large 27‐Mbp Mesoamerican introgression on chromosome Pv02. The largest introgressions in varieties from the Mesoamerican gene pool were found spanning the complete chromosome Pv10 of ‘TARS‐VR‐7S’ and in the interspecific advanced line ‘MIB 778’ containing a 22‐Mbp introgression in the upper arm of chromosome Pv06 (Supplemental Table S3).

A very interesting introgression of a Mesoamerican haplotype covering 24 Mbp of the pericentromeric region of chromosome Pv08 was identified within the Andean cultivar G19833. This introgression was consistently identified in the Andean varieties G4627, G24705, and G5686. This outcome is surprising because these landraces have been considered as typical Andean accessions because of their phenotypic behavior; in particular, G19833 was selected to build the common bean Andean reference genome (Schmutz et al., 2014). However, we could identify other Mesoamerican introgressions of more than 1 Mbp in chromosomes Pv04, Pv07, and Pv11 in this variety. The other three varieties sharing the introgression in chromosome Pv08 also contained other large introgressions, mainly in chromosome Pv04, in which the variety G5686 harbored a completely Mesoamerican haplotype.

Using a similar analysis on the dataset containing *P. coccineus* and *P. acutifolius*, we corroborated the DNA introgression from *P. acutifolius* to *P. vulgaris* in the interspecific cross‐derived Vulgaris Acutifolious Xantomonas (VAX) lines developed at CIAT (Beebe, 2012; Singh & Muñoz, 1999). The interspecific lines ‘VAX 3’, ‘VAX 4’, and ‘VAX 6’ contained a common 500‐kbp *P. acutifolius* haplotype block on chromosome Pv08 (Supplemental Table S4). This introgression was also found in the advanced line ‘INB 841’, which is also known to be derived from an interspecific cross with *P. acutifolius*. The advanced line ‘ALB 213’ shows three regions of introgression with *P. coccineus* on chromosomes Pv08, Pv09, and Pv11. This is expected because ALB 213 is the only advanced line known to be derived from an interspecific cross with *P. coccineus* within the sequenced accessions. Interestingly, the Mesoamerican accessions G10474 and Mexico 54 also contain a 500‐kbp introgression segment at Pv09 that was found in ALB 213. The common *P. acutifolius* introgression found on chromosome Pv08 is very important for breeding purposes because alleles from *P. acutifolius* at this locus [tagged by the SU91 marker (Miklas et al., 2006)] provide VAX lines with a level of resistance to common bacterial blight that is not achieved by other *P. vulgaris* cultivars. Hence, development of SNP markers to track and transfer *P. acutifolius* haplotypes to other genetic backgrounds can be focused on this region that was consistently introgressed in the VAX 4, ‘VAX 5’, and VAX 6 lines.

By use of a SNP that is unique for *P. acutifolius* haplotypes in this region, a new marker was developed (CBBChr08_TA_59475020) with KASP technology (LGC Genomics) (Table 1). This SNP‐based marker produced identical results to the previously developed SCAR marker SU91 (Miklas et al., 2006) in the evaluated panel distinguishing *P. acutifolius* and *P. vulgaris* alleles at this locus. Another SNP‐based marker designed in physical proximity to the SCAR marker SAP6, located on chromosome Pv10, showed a smaller correlation with the phenotype, again similar to the preceding SCAR. These new CBB markers offer a platform advantage over previous markers for genotyping on scalable gel‐free systems that allow higher throughput, faster, and more cost‐effective genotyping.

**Table 1:** Evaluation of molecular markers for disease resistance in *Phaseolus* accessions. The physical locations of the markers are available at Supplemental Table S5.

Marker ID	Angular leaf spot																				Common bacterial blight					
	Marker50	MAS_ALS4b	ALSChr04_GC_43800347	MAS_ALS5a	Marker31	MAS_ALS5b	ALSChr08_CT_57798588	PF5	CB_00451	MAS_ALS10a	Sc267437	Sc267437_HRM	ALSChr09_TA_17203795	Marker33	ALSChr09_CT_17294365	ALSChr09_CT_17299731	MAS_ALS10b	Marker17	MAS_ALS10c	ALS evaluation (location)‡, §sss	SU91	CBBChr08_TA_59475020	SAP6	CBBChr10_CG_40020540	CBB evaluation (Xc123)‡, §	CBB evaluation (Xcf631)‡, §
Marker type†	Tms	K	K	K	Tms	K	K	S	Tms	K	Tms	HRM	K	Tms	K	K	K	Tms	K		S	K	S	K		
G10474	T:T	**T:T** **#**	**G:G**	A:A ††	A:A	**T:T**	**T:T**	+	**A:A**	G:G	G:G	G:G	**T:T**	A:A	**?**	**C:C**	A:A	A:A	**C:C**	1 (Q)	–	T:T	–	G:G	9	9
G4691	T:T	T:T	G:G	A:A	A:A	T:T	T:T	+	G:G	G:G	G:G	G:G	T:T	A:A	?	C:C	A:A	A:A	C:C	2 (Q)	–	T:T	+	C:C	9	9
G855	T:T	T:T	G:G	A:A	A:A	T:T	C:C	+	G:G	T:T	T:T	T:T	T:T	G:G	C:C	C:C	A:A	A:A	C:C	2 (D1)	–	T:T	+	C:C	9	9
G18970	T:T	T:T	G:G	A:A	A:A	T:T	C:C	+	A:A	G:G	G:G	G:G	T:T	G:G	C:C	C:C	A:A	A:A	C:C	2 (D1)	–	T:T	–	G:G	9	9
G10909	G:G	T:T	G:G	A:A	A:A	T:T	C:C	+	G:G	G:G	G:G	G:G	T:T	G:G	?	C:C	A:A	A:A	C:C	3 (Q)	–	T:T	–	G:G	9	9
G5686	G:G	C:C	C:C	A:A	A:A	C:C	C:C	**–**	G:G	T:T	T:T	T:T	A:A	A:A	T:T	T:T	C:C	C:C	T:T	3 (Q)	–	T:T	–	G:G	9	9
MAB 300	?	T:T	G:G	A:A	A:A	T:T	C:C	+	G:G	T:T	T:T	T:T	T:T	A:A	C:C	C:C	A:A	A:A	C:C	3 (Q)	–	T:T	+	G:G	9	9
SEL 1481	G:G	T:T	G:G	A:A	A:A	T:T	C:C	+	G:G	T:T	T:T	T:T	T:T	A:A	C:C	C:C	C:C	C:C	C:C	3 (GH)	–	T:T	–	G:G	9	9
AND 277	G:G	C:C	C:C	A:A	A:A	T:T	C:C	–	G:G	T:T	T:T	T:T	T:T	A:A	C:C	C:C	C:C	C:C	C:C	4 (Q)	–	T:T	–	G:G	6	6
Mexico 54	T:T	T:T	G:G	A:A	A:A	T:T	C:C	–	G:G	G:G	G:G	G:G	T:T	G:G	?	C:C	?	A:A	C:C	4 (D1)	–	T:T	+	C:C		
G5653	T:T	T:T	G:G	A:A	A:A	T:T	C:C	–	G:G	G:G	G:G	G:G	T:T	G:G	C:C	C:C	A:A	A:A	C:C	4 (D1)	–	T:T	–	G:G	9	9
MBC 35	G:G	T:T	G:G	A:A	A:A	T:T	C:C	+	G:G	T:T	T:T	T:T	T:T	A:A	C:C	C:C	C:C	C:C	C:C	5 (GH)	–	T:T	–	G:G	9	9
SEL 1478	G:G	C:C	C:C	A:A	A:A	T:T	C:C	–	G:G	T:T	T:T	T:T	T:T	A:A	C:C	C:C	C:C	C:C	C:C	5 (GH)	–	T:T	–	G:G	9	9
MBC 33	G:G	T:T	G:G	G:G	G:G	T:T	C:C	–	G:G	T:T	T:T	T:T	T:T	A:A	C:C	C:C	C:C	C:C	C:C	5 (GH)	–	T:T	–	G:G	9	9
MBC 39	G:G	T:T	G:G	A:A	A:A	T:T	C:C	–	G:G	T:T	T:T	T:T	T:T	A:A	C:C	C:C	C:C	C:C	C:C	5 (GH)	–	T:T	–	G:G	9	9
NUA 56	T:T	T:T	G:G	A:A	A:A	T:T	C:C	–	G:G	T:T	T:T	T:T	T:T	A:A	C:C	C:C	C:C	C:C	C:C	6 (GH)	–	T:T	–	G:G	7	6
G1805	T:T	T:T	G:G	A:A	A:A	T:T	C:C	+	A:A	T:T	T:T	T:T	T:T	G:G	C:C	C:C	A:A	A:A	C:C	6 (D1)	–	T:T	–	G:G	9	9
G14519	T:T	T:T	G:G	A:A	A:A	T:T	C:C	+	A:A	G:G	G:G	G:G	T:T	G:G	C:C	C:C	A:A	A:A	C:C	7 (GH)	–	T:T	+	C:C	9	9
SEL 1475	?	T:T	G:G	A:A	A:A	T:T	C:C	–	A:A	G:T	G:T	?	T:T	G:G	C:C	C:C	A:A	A:A	C:C	7 (GH)	–	T:T	–	G:G	9	9
SEL 1476	G:G	T:T	G:G	G:G	G:G	T:T	C:C	–	G:G	T:T	T:T	T:T	T:T	A:A	C:C	C:C	C:C	C:C	C:C	7 (GH)	–	T:T	–	G:G	9	9
SEL 1477	G:G	T:T	G:G	G:G	G:G	T:T	C:C	–	G:G	T:T	T:T	T:T	T:T	A:A	C:C	C:C	C:C	C:C	C:C	7 (GH)	–	T:T	–	G:G	9	9
G21242	G:G	?	?	G:G	G:G	T:T	C:C	+	A:A	?	G:G	G:G	T:T	A:A	C:C	C:C	A:A	A:A	C:C	8 (GH)	–	T:T	–	C:C	9	9
G23823E	T:T	T:T	G:G	A:A	A:A	T:T	C:C	+	G:G	T:T	T:T	T:T	T:T	G:G	C:C	C:C	A:A	A:A	C:C		–	T:T	+	G:G	9	9
MBC 7	G:G	T:T	G:G	A:A	A:A	T:T	C:C	+	G:G	T:T	T:T	T:T	T:T	A:A	C:C	C:C	A:A	A:A	C:C	8 (GH)	–	T:T	+	C:C	9	9
MBC 26	G:G	T:T	G:G	G:G	G:G	T:T	C:C	–	G:G	T:T	T:T	T:T	T:T	A:A	C:C	C:C	C:C	C:C	C:C	8 (GH)	–	T:T	–	G:G	9	9
MBC 28	G:G	T:T	G:G	G:G	G:G	T:T	C:C	–	G:G	T:T	T:T	T:T	T:T	A:A	C:C	C:C	C:C	C:C	C:C	8 (GH)	–	T:T	–	G:G	9	9
MBC 34	G:G	T:T	G:G	G:G	G:G	T:T	C:C	–	G:G	T:T	T:T	T:T	T:T	A:A	C:C	C:C	C:C	C:C	C:C	8 (GH)	–	T:T	+	G:G	9	9
Sprite	T:T	T:T	G:G	G:G	G:G	T:T	C:C	–	G:G	T:T	T:T	T:T	T:T	G:G	C:C	C:C	A:A	A:A	C:C	8 (D1)	–	T:T	–	G:G	9	9
BAT 93	T:T	T:T	G:G	A:A	A:A	T:T	C:C	+	G:G	T:T	T:T	T:T	T:T	A:A	C:C	C:C	A:A	A:A	C:C	6 (D1)	–	T:T	–	G:G	5	5
XAN 112	T:T	T:T	G:G	A:A	?	T:T	C:C	+	?	G:G	G:T	G:G	T:T	A:A	C:C	C:C	A:A	A:A	C:C	7 (D1)	–	T:T	+	C:C		
Jules	T:T	T:T	G:G	A:A	A:A	T:T	C:C	+	G:G	G:G	G:G	G:G	T:T	G:G	C:C	C:C	A:A	A:A	C:C		–	T:T	+	C:C	5	5
VAX 2	?	T:T	G:G	A:A	A:A	T:T	C:C	–	G:G	T:T	T:T	T:T	T:T	A:A	C:C	C:C	A:A	A:A	C:C	7 (D2)	–	T:T	+	C:C	4	4
VAX 1	T:T	T:T	G:G	A:A	A:A	T:T	C:C	–	G:G	T:T	T:T	T:T	T:T	A:A	C:C	C:C	A:A	A:A	C:C	7 (D2)	–	T:T	+	C:C	2	2
SEL 1309	T:T	T:T	G:G	A:A	A:A	T:T	C:C	–	G:G	T:T	T:T	T:T	T:T	G:G	C:C	C:C	A:A	A:A	C:C		+	A:A	–	G:G		
XAN 159	T:T	T:T	G:G	G:A	G:G	T:T	C:C	+	G:G	T:T	T:T	T:T	T:T	G:G	C:C	C:C	C:C	C:C	C:C	5 (D1)	+	A:A	–	G:G	5	6
G40001	T:T	?	?	G:G	G:G	T:T	?	?	A:A	G:G	G:G	G:G	?	G:G	?	?	A:A	A:A	?		+	A:A	–	C:C	2	2
VAX 5	T:T	T:T	G:G	A:A	A:A	T:T	C:C	–	G:G	T:T	T:T	T:T	T:T	A:A	C:C	C:C	A:A	A:A	C:C		+	A:A	+	C:C	2	2
VAX 3	?	T:T	G:G	A:A	A:A	T:T	C:C	–	G:G	T:T	T:T	T:T	T:T	A:A	C:C	C:C	A:A	A:A	C:C		+	A:A	+	C:C	1	2
VAX 4	T:T	T:T	G:G	A:A	A:A	T:T	C:C	–	G:G	T:T	T:T	T:T	T:T	A:A	C:C	C:C	A:A	A:A	C:C		+	A:A	+	C:C	1	1
VAX 6	T:T	T:T	G:G	A:A	A:A	T:T	C:C	–	G:G	T:T	T:T	T:T	T:T	A:A	C:C	C:C	A:A	A:A	C:C	7 (D2)	+	A:A	+	C:C	1	1

† The marker types used were melting temperature shift (Tms), KASP (K), sequence characterized amplified region (S), and high‐resolution melting point (HRM).

‡ Angular leaf spot (ALS) and common bacterial blight (CBB) evaluations: resistant (1–3), intermediate (4–6) or susceptible (7–9).

§ Angular leaf spot evaluation conditions in the field. Q, Quilichao field site, inoculated with the isolates Pg 331–1 and Pg 347 (race 63–63); D1: Darien field site, natural inoculation; D2: Darien field site, inoculated with a mixture of ALS pathotypes; GH: greenhouse isolate Pg353 (race 63–47). CBB was evaluated in the greenhouse.

# Unique single nucleotide polymorphisms for ALS resistance sources G10474 and G5686 are shown in bold.

†† Disease resistance associated alleles are underlined.

### Estimation of Nuclear DNA Content for Cultivated *Phaseolus* Species

**Fig. 3:**
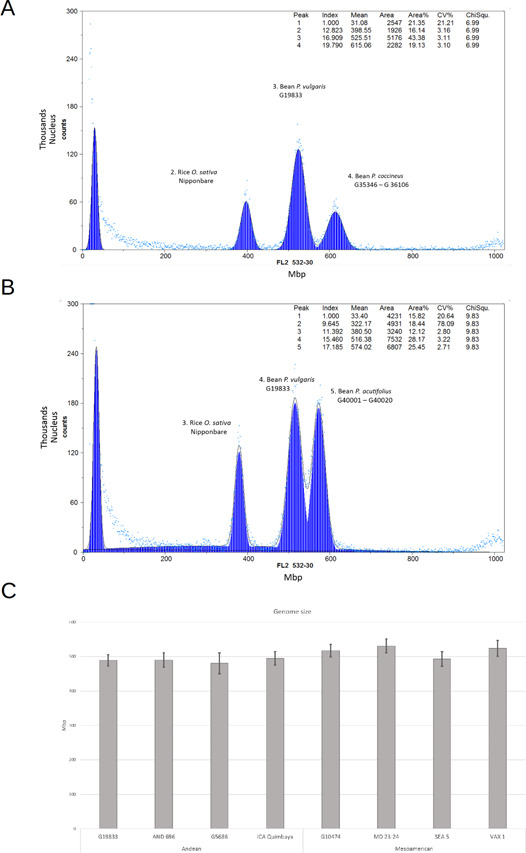
Genome size of *Phaseolus* species and *P. vulgaris* genotypes determined by flow‐cytometry. The flow cytometry charts compare the DNA content of nuclei extracted from G19833 and two accessions of *P. coccineus* (A) and two accessions of *P. acutifolius* (B). The DNA content of *Oryza sativa* cv. Nipponbare (395 Mbp) was used to calibrate the amount of DNA. (C) Genome sizes of *P. vulgaris* samples of the Andean and Mesoamerican gene pools normalized with the reference genome G19833. Error bars represent the SD of the sample distribution.

To identify correlations between genome size and mapping rates, nuclear DNA content was analyzed. Measurements with the PARTEC flow‐cell cytometer (Sysmex Partec GmbH) revealed larger genome sizes for *P. coccineus* and *P. acutifolius* than for *P. vulgaris*. A genome size of 525 Mbp was estimated for G19833, the *P. vulgaris* accession chosen to obtain the Andean bean reference genome (Schmutz et al., 2014). This is only 12 Mbp smaller than the total length of the current reference assembly (version 2.1), which is 537 Mbp. The genome sizes of *P. coccineus* lines were estimated as 620 Mbp (Fig. 3A) and those of *P. acutifolius* at 575 Mbp (Fig. 3B). This may partially explain the lower read alignment rates that we obtained in these two species related to *P. vulgaris* included in this study. Additional DNA is likely to contain expansions of repetitive elements as well as unique genomic regions with novel genes. Comparing the Mesoamerican and Andean lines within *P. vulgaris* resulted in a slightly but significantly higher nuclear content of Mesoamerican genotypes (*t*‐test: *p* = 0.0035) (Fig. 3C). Although the difference in DNA content within *P. vulgaris* is not as large as that observed in the comparisons with the other two congeneric species, genes within the additional DNA content of Mesoamerican varieties may not be represented in the Andean reference genome. Small genome size variations among the Andean and Mesoamerican samples analyzed may highlight the genomic diversity and potentially hidden genetic information within *P. vulgaris* species.

### Discovery and Characterization of CNVs

The availability of WGS data allowed us not only to identify SNPs but also to apply different techniques to identify structural variations. We performed the RD analysis available in NGSEP to identify CNVs in the individual *P. vulgaris* accessions sequenced in this study. Polymorphisms were predicted that deviated from the reference copy number of two copies per diploid genome. On average, 2959 CNVs per sample had deletion alleles with predicted copy numbers of one (heterozygous deletion) or zero (homozygous deletion) and, on average, 12,451 CNVs showed a predicted number of copies greater than two (duplication alleles). Deletion CNVs had a mean length of 6 kbp, amounting to 17.7 Mbp of deleted sequence per genome, on average, whereas duplications showed a mean length of 12.5 kbp, representing 246.9 Mbp of duplicated sequence per genome on average. This is an expected outcome of the RD analysis executed in this experiment because three alignments were kept for reads aligning to multiple sites, which inflated the average RD in repetitive regions of the genome [see a more elaborate discussion about RD analysis in Duitama et al. (2014)]. If we considered only the 228 Mbp of the nonrepetitive reference genome, deletions and duplications span, on average, 6.3 and 25.9 Mbp, respectively. Predicted duplications for the two accessions (Midas and SXB 412) cover an abnormally high amount of the nonrepetitive portion of the reference genome (Supplemental Fig. S3). Considering that this could be the effect of false positive calls, we decided to remove these two accessions for the rest of the analysis.

Clustering of the predicted CNV sites in the 35 remaining samples resulted in a raw dataset of 133,804 nonredundant CNVs. Analogous to the process used to genotype the SNPs, copy number of each nonredundant CNV was predicted for each sample based on the RD within the CNV compared with the average RD across the genome for that sample and taking into account its variance (see the Materials and Methods for details). A dataset of 3.4 million CNV genotype calls was assembled with 26.6%of the data missing. On the basis of this percentage, the samples can be clustered in two groups, the first having about 20% missing data and the second having about 35% missing data (Supplemental Fig. S4). With the exception of the Mesoamerican accessions BAT 93, RWR 719, TARS‐VR‐7s, and VAX 4, a higher percentage of missing data can be explained by the lower average RD.

**Fig. 4:**
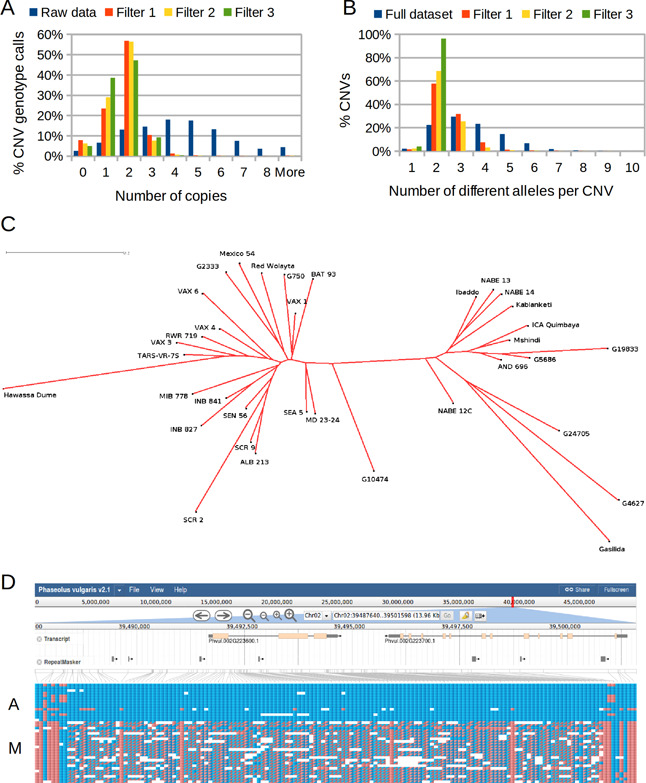
Patterns of copy number variation (CNV) variability in *P. vulgaris* genotypes. The summary of the analysis of CNVs in the bean genome is based on the whole‐genome sequencing (WGS) data of 35 accessions sequenced in this study. (A) Number of alleles per CNV and (B) average number of copies predicted at each CNV call, calculated from the raw dataset and the datasets filtering out repetitive regions (Filter 1), genotyped in at least 30 samples (Filter 2), and showing at most two alleles (Filter 3). (C) Dendrogram based on predictions of copy number for 669 duplications in nonrepetitive regions of the genome. (D) Transcript annotations (orange tracks), annotation of repetitive elements (gray track), and single nucleotide polymorphism (SNP) genotype calls over the 37 varieties sequenced in this study (blue and red lower panel) for the region covering the genes *Phvul.002G223600* and *Phvul.002G223700*. The read depth (RD) analysis consistently predicts a duplication in the Mesoamerican lines within these regions. Homozygous calls to the reference allele are shown in blue, whereas homozygous calls to an alternative allele are shown in red. Heterozygous calls are shown as two red and blue triangles. A, Andean lines; M, Mesoamerican lines.

Similar to the filtering process for SNP genotype calls, filters for (i) repetitive elements, (ii) the number of individuals genotyped, and (iii) number of different alleles were applied, leaving 18,555, 3871, and 2056 CNVs respectively (see the Materials and Methods for details). Figure 4 shows how the predicted number of copies per CNV per accession and the number of different alleles per CNV vary when these filters were applied. Because most of the predicted CNVs in the raw dataset colocated with repetitive regions of the genome, once the filter of intersection with repetitive regions (Filter 1) is applied, most of the predictions of high copy number (>3) present in the raw dataset disappeared and the reference copy number of two became predominant (Fig. 4A). Likewise, the percentage of duplication events reduced from 75 to 19%. Regarding the number of alleles observed per CNV, Filters 1 and 2 raised the frequency of biallelic CNVs from 22 to 58 and 68% respectively before the explicit filter to retain biallelic CNVs is applied (Fig. 4B). Furthermore, a high number of calls with a copy number of 1 and a copy number of 3 were predicted, which may indicate a problem differentiating the copy number calls 0 to 1 and 3 to 4, respectively.

If we considered the 669 duplications and the 2287 deletions of the Filter 2 dataset separately, neighbor‐joining dendrograms were constructed from the distances, calculated as the differences between predicted copy numbers for each pair of samples. The 35 samples were clustered into two groups consistent with the results obtained with the database of SNPs (Fig. 4C). Within two biallelic duplications, predictions of at least three were are observed for every Mesoamerican accession and none for the Andean accessions. The same analysis using deletions also allowed us to reconstruct the expected dendrogram (Supplemental Fig. S5) and revealed 299 regions with biallelic deletion events exclusively predicted for every Mesoamerican accession. This result serves as an indirect validation of the accuracy of predicting regions with abnormal copy numbers.

A total of 602 (90%) of the Filter 2 duplications and 1060 (46%) of the Filter 2 deletions overlapped and even sometimes completely covered the annotated genes. The number of different annotated genes in nonrepetitive regions potentially affected by CNVs is 558 for duplications and 959 for deletions. Figure 4D features SNP genotype calls for the accessions sequenced in this study within a region in chromosome Pv02 covering the genes *Phvul.002G223600* and *Phvul.002G223700*. For one duplication covering this region, four copies were predicted in almost all Mesoamerican accessions (with the exception of G10474), whereas a reference copy number (2) was predicted for the Andean accessions. Consistent with the predictions of a high copy number, the Mesoamerican accessions (except for G10474) showed a large cluster of heterogeneous SNP calls in this region. The lack of large repetitive structures within this region (the gray track in Fig. 4D) suggests that the heterogeneous genotypes are likely to be produced by differences between the copies of the duplicated genes, which were present only in Mesoamerican accessions and were consequently not represented in the reference genome.

### Molecular Markers for Resistance to ALS

Molecular markers tagging valuable loci in recombinant inbred line (RIL) populations are often not widely applicable for MAS because they are not specifically polymorphic in different backgrounds of breeding germplasm. Using the WGS dataset presented here, we developed molecular markers with improved specificity and polymorphism for five reported ALS resistance loci. The SCAR marker PF5 on chromosome Pv08 is associated with one of the strongest ALS resistance genes, originating from the landrace G10474 (Mahuku et al., 2004). Single nucleotide polymorphisms in the vicinity of PF5 were selected to develop new molecular markers, either from the BARCBean chip (Song et al., 2015) or from the WGS dataset of CIAT lines, selecting SNPs that are unique to the resistance source G10474. These markers were evaluated in a panel of 40 genotypes containing known resistance sources for ALS and CBB (Table 1 and Supplemental Table S5). The WGS‐selected marker ALSChr08_CT_57798588 showed superior specificity, only tagging the resistance source G10474 and also G4691. For other markers on chromosome Pv08, on the other hand, the allele found to be associated with resistance in the original RIL study appears in many susceptible genotypes. Similarly, the WGS data set was applied to select new SNPs adjacent to four ALS resistance loci reported from the Andean genotype G5686 (Keller et al., 2015). Previously reported SNP markers (Marker 50, Marker 31, Marker 33, and Marker 17) are expected to be polymorphic between the parental lines G5686 and ‘Sprite’ used in that study, but did not specifically distinguish the resistance source in this panel. New markers on chromosome Pv04 (ALSChr04_GC_43800347 and the previously reported MAS_ALS4b) more specifically tag the resistance sources G5686, ‘AND 277’, and ‘SEL 1481’, whereas new markers linked to the resistance loci on chromosomes Pv05, Pv09, and Pv10 differentiate G5686 from all other genotypes in the panel. The new SNP‐based markers are more likely to effectively tag the resistance gene in different crosses.

In addition to identifying markers with improved specificity for all five ALS loci, marker development for the same SNPs was demonstrated on different platforms. Redundant markers are indicated by the identical positions of markers on chromosomes Pv05, Pv08, and Pv10 (Table 1 and Supplemental Table S5). As an example, the melting temperature shift marker (sc267437), the HRM marker (sc267437_HRM), and a KASP assay (LGC Genomics), genotyped by a commercial genotyping service (MAS_ALS10a), which were developed from the same SNP on chromosome Pv08, produced the same results. The choice of different genotyping technologies provides more flexibility to select the best marker platform suited to laboratory resources at different institutions and in different parts of the world. The SNP markers selected in the relatively small WGS dataset showed superior specificity in the tested panel for all five ALS resistance loci and may therefore also work better for MAS in different breeding germplasms.

## Discussion

In this work, we describe the genomic characterization of breeding germplasm from diverse breeding programs, using the unprecedented depths of information provided by WGS data. In a joint effort by CIAT and UC Davis, a WGS of 35 common bean lines and two accessions of related species was performed. These sequences, together with other published datasets, were analyzed jointly. The specific lines were selected because of their superior agronomic traits such as yield, grain type, resistance to different biotic and abiotic stresses, and their current use in crossing programs for varietal development in North and South America, as well as in Eastern and Southern Africa. This spans most regions of recent active breeding efforts, excluding only the Brazilian efforts, where national policies inhibit germplasm exchange with other countries, and the largely unknown efforts in Northern India and China, which are not well connected with the bean community.

Analysis of several genetic characteristics revealed information that enhances the understanding and future uses of this germplasm. Mapping of introgressions revealed the partly unknown history of these lines, which is relevant in planning future crosses. Diversity evaluation between long‐standing breeding programs allows us to see the breadth and potential overlaps among breeding gene pools. Whole‐genome sequencing also delivers directly usable molecular breeding tools, providing polymorphic markers the tag different genes with high specificity for the donor genotypes. Together, this information will be used to increase the efficiency of breeding programs for both commercial production in the Americas and the largely smallholder farming systems in Africa.

### Intra‐ and Interspecific Introgressions

This study shows accurate chromosomal positioning of intraspecific, inter–gene pool introgressions within the Andean and Mesoamerican backgrounds, following techniques similar to those used to find extensive introgression events in rice (Duitama et al., 2015) and to identify introgression of *Manihot glaziovii* Müll. Arg. haplotypes assumed to confer disease resistance to African cassava (*Manihot esculenta* Crantz) accessions (Bredeson et al., 2016). Recently, Ferreira et al. (2016) identified introgressions in 16 common bean genotypes that resulted from a backcrossing program for several disease resistance loci into breeding germplasm. On the basis of genotyping‐by‐sequencing data, between 0.33 and 6.88% of introgressions were detected in each genome, visualizing the results of the breeding process and confirming known resistance loci for BCMV and anthracnose. Previous studies reporting inter–gene pool introgression events in common bean (Blair et al., 2010, Muñoz et al., 2014) were based on amplified fragment length polymorphism and simple sequence repeat markers; hence, they could not report the site and size of introgression events with the same precision as can be done with WGS data. The extent of the introgressions identified in this study was larger than expected and included surprising cases such as a large Mesoamerican segment covering the pericentromeric region of chromosome Pv08 in G19833, which is considered a typical Andean landrace. This may indicate more ancient seed movement and admixture than commonly assumed.

The lines showing the same Mesoamerican introgression event as in G19833 (i.e., G4627, G24705, and G5686) share a distribution on the eastern slope of the Andes (Colombia, Ecuador, and Peru), suggesting seed exchanges within that region. Evidence for this was noted by Islam et al. (2004) in an analysis of the effects of inter–gene pool introgression on agronomic traits in landraces from the Andes. A similar observation was made for the A4d haplotype of the *PvTFL1y* gene (Kwak et al., 2012), which is responsible for one of the naturally occurring determinacy genotypes in Andean domesticated *P. vulgaris*. Accessions G4647, G24705, G24800 (all in Colombia), and G2686 (Peru) from the study by Kwak and Gepts (2009) all share the A4d haplotype and are similarly distributed on the eastern slope of the Andes. This is a geographical region in which accessions from both gene pools were widely grown in pre‐Colombian times and where genetic exchange has been documented previously (Gepts et al., 1986). It remains to be explained why this particular sequence would have been introgressed repeatedly or, alternatively, why a unique introgression would have been conserved. From a genomics perspective, this finding suggests that an additional Andean accession should be sequenced to establish an Andean reference genome to reveal Andean haplotype segments that are currently not visible because of the extensive introgression events identified in G19833.

The detection of the *P. acutifolius* haplotypes present in the VAX lines confirms that introgression of haplotypes from outgroup species into common bean is not only feasible but it is also useful for introducing alleles conferring desired traits such as resistance to CBB. Single nucleotide polymorphism markers developed within the introgressed region correlate with the well‐known SCAR marker SU91, which tags CBB resistance, and can then be used to track *P. acutifolius* haplotypes in future crosses.

### Genome Size Variation

Genome size estimations of *P. acutifolius* and *P. coccineus* revealed the larger genomic content of these species compared with that of G19833, with a clearer signal than previous measurements that found the same correlation. Previous reports of the *P. vulgaris* genome size by Nagl and Treviranus (1995) (1.40–1.53 pg, ∼719 Mbp) and Barow and Meister (2003) (1.58 pg, ∼780 Mbp) are variable and suggest a larger size. A comparison of the measurement for G19833 against the total length of the current assembly (version 2.1) suggests that our estimations of genome size are more accurate than previous reports. The data in this work also indicate that Mesoamerican types tend to have a larger genome than Andean types, partially explaining the reduced alignment rates observed between the sequenced materials and the Andean reference genome, in addition to phylogenetic divergence. A larger germplasm set will have to be evaluated to confirm this observation. Measurements of DNA content for Mesoamerican varieties are consistent with the total assembly length of 550 Mbp reported for the Mesoamerican accession BAT 93 (Vlasova et al., 2016), which is 13 Mbp larger than the version 2.1 assembly length of G19833 (Phytozome, 2017). Unaligned reads may represent gene pool–specific genomic segments not represented in the current reference. A similar situation has been observed in *O. sativa*, comparing de novo assemblies of *indica* varieties against the current reference (i.e., the temperate *japonica* cultivar Nipponbare) (Schatz et al., 2014).

Another cause of large differences in genome size is the occurrence of large deletion or duplication events in the evolutionary history of the species. A phylogenetic analysis of paralog gene families identified in the BAT 93 assembly reported more than 5000 genes that were possibly involved in recent duplication events (Vlasova et al., 2016). The number of copies (or dosage) of segments involved in these events across a population defines alleles for CNVs. The analysis of RD patterns across the genome from WGS data at sufficient average coverage allowed us to identify and genotype more than 100,000 CNVs within the accessions sequenced in this study. As expected, most of the duplication events were already represented in the reference genome, either as tandem or as interspersed duplications. Separate clustering analysis based on biallelic deletions and duplications in nonrepetitive regions coincided with the clustering obtained with SNP markers, which indicates that the predictions of the number of copies obtained from the RD analysis were generally accurate.

More than 100 CNVs distinguished Andean and Mesoamerican samples and spanned large portions of coding regions. This suggests that CNVs contribute to the overall difference in DNA content between Andean and Mesoamerican varieties and also that they can be relevant as genomic drivers of trait variation between these populations. Given that a complete de novo reconstruction of the genomes of more than a handful of individual accessions is still a major endeavor, bioinformatic analysis of short‐read sequencing data is still a cost‐effective alternative to identifying a large number of structural events across populations. However, the RD analysis is generally biased by the cultivar used to build the reference genome. The recent assembly of the Mesoamerican genome BAT 93 (Vlasova et al., 2016) and high‐quality future assemblies of accessions of related species that use emerging techniques such as PacBio would be desirable to perform a more comprehensive analysis of structural variation, both within *P. vulgaris* and between *P. vulgaris* and related species.

### Sequence Variation as a Genomic Resource

Similar WGS activities have been recently published for crops such as rice (Huang et al., 2012; Duitama et al., 2015), sorghum (Mace et al., 2013), bell pepper (Qin et al., 2014), and tomato (Lin et al., 2014). Combining the sequencing data obtained in this study with data generated from previous work (Schmutz et al., 2014; Vlasova et al., 2016; Song et al., 2015), we developed a genomic variation dataset including more than 20 million variants within *P. vulgaris* that increased to more than 40 million variants when we included variation against two other domesticated *Phaseolus* species, *P. coccineus* and *P. acutifolius*. Clustering based on genotype calls of these variants correlated well with the known relatedness to the Andean reference (Kwak & Gepts, 2009). Comparable studies reported a total of 7.9 million SNPs based on low‐coverage WGS of 1083 diverse *O. sativa* varieties and 446 *Oryza rufipogon* Griff. accessions (Huang et al., 2012). Likewise 9.8 million SNPs were detected in 18 cultivars and two semiwild or wild accessions in bell pepper (Qin et al., 2014) and 4.9 million SNPs were identified in *S. bicolor* populations (Mace et al., 2013). The main reason for the larger number of variants reported in this study is that repetitive regions were not systematically eliminated from the analysis, as it was performed in the pipelines of other analyses. Stringent filtering for repetitive regions based on published reference information, regions of nonunique read alignments, and regions with evidence of CNVs reduced the number of polymorphisms by >70%. Filtering for all repetitive regions eliminated variants that appeared to be caused by differences between repetitive elements, albeit, at the cost of reducing the actual size of the analyzed genome to about 200 Mbp of unique sequence. Although the filtered set was less error‐prone and more suitable for marker design, genomic analysis or detailed analysis of specific regions may require the complete unfiltered set to be used.

Single nucleotide polymorphism‐based clustering of the accessions analyzed in this study largely coincided with their expected background population. In comparison with other studies that investigated the broader diversity of *Phaseolus* species and races (Rendón‐Anaya et al., 2017), this study reflects the work of different breeding programs in several continents. Within Mesoamerican gene pool lines, the Durango lines cluster as the most distinct group. Generally, North American varieties of the race Durango (Fig. 1C, Group III), the race Mesoamerica (Group I), and the Andean gene pool (Group V) cluster largely in distinct groups, indicating a narrow genetic diversity that was probably caused by the strict grain class specifications required by the market. This is less pronounced in South American breeding material. African varieties are scattered over all groups, in line with the absence of a dedicated African breeding program that would establish a separate breeding gene pool. This also reflects the fact that some African breeding materials have often been imported from several established breeding centers, mainly in the Americas. Taken together, it appears to be more difficult to infuse new genetic variability into breeding programs with very strict industry‐dictated grain type targets such as those in North America. Global germplasm exchange was active in past decades, but according to these data, not much admixture has been taken up into new varieties. Less regulated markets in African regions adopt more variable germplasm, which makes it easier from the perspective of the breeders to fully use the observed introgressions of alien haplotypes that are an important driver of variability.

### Application to Marker Identification

Next‐generation sequencing resources, as developed in this study, are expected to be used in different ways to develop molecular tools for applied breeding. A set of WGS genotyping data facilitates the selection of diagnostic polymorphisms, SNPs, and indels, distinguishing desired genotypes not only in specific crosses but, for most cases, also in additional complex populations and panels. Here we demonstrate polymorphism selection and SNP‐based marker design to replace ALS resistance markers that were identified in specific RIL populations. A multitude of RIL studies have been published (Gepts et al., 2008) but application of trait‐associated markers in MAS has been low, as the reported markers often do not specifically tag the source of the valuable alleles in different genetic backgrounds (Table 1). By using the CIAT WGS data, we identified improved markers for five reported ALS resistance loci on chromosomes Pv04, Pv05, Pv08, Pv09, and Pv10. These new markers showed a higher specificity to tag the resistance sources in a test panel than previously reported markers. This observation raises the question of the size of the WGS set that is required to identify widely applicable markers. This issue needs to be confirmed with a larger sequenced sample. As an example for marker application, Namayanja et al. (2006) reported the validation efforts of two markers linked to the *Phg‐2* gene (the PF5 marker). OPE04 was considered to be useful, whereas OPN02 failed because it was not polymorphic within breeding populations. New, highly source‐specific markers such as ALSChr08_CT_57798588 will have much lower rates of failure and are expected to work for MAS in other populations derived from the same base haplotypes.

We verified that the genotyping of selected SNPs was robust across different technologies and commercially available genotyping platforms such as KASP assays (LGC Genomics), melting temperature shift markers, or HRM markers. This provides flexibility for laboratories to choose the marker technology suited to their needs and resources. Single nucleotide polymorphism‐based markers for gel‐free systems are now available to replace major effect SCAR markers for CBB (SU91, SAP6) and ALS (PF5). The biallelic indel polymorphisms detected in this work can also be selected for marker design for evaluation in low‐tech labs.

A WGS dataset is also valuable for increasing marker density and fine mapping. The 13,261 SNPs differentiating the Andean and Mesoamerican gene pools (Andean vs Mesoamerican SNP list) are expected to be largely polymorphic for any of the commonly used inter–gene pool RIL populations. For example, Oblessuc et al. (2013) described a major effort to increase marker density for an ALS resistance region resulting in a marker list still presenting long gaps (>1 Mbp). The Andean‐Mesoamerican SNP list presents a useful tool for increasing marker density in such cases. Whole‐genome resequencing of the parental lines of mapping populations provides additional value, as it supplies saturated polymorphism density and it technically allows us to see all polymorphisms between genotypes. Whole‐genome resequencing reveals that only a low percentage of genes in the available RIL populations actually harbor nonsynonymous polymorphisms (‘SEA 5’ × ‘MD 23–24’, 18%; ‘AND 696’ × G19833, 15%, ‘AFR298’ × ‘VAX 1’ 61%). As genetic studies often result in long lists of candidate genes, this information is a powerful tool for narrowing down the number of likely candidate genes.

The availability of a reference genome for common bean, the continuous reductions in sequencing costs, and the development of different methods for bioinformatic analysis of the sequencing data provide a solid infrastructure to accelerate the research needed to achieve a deep understanding of bean genomic variability. A joint effort across the bean genomics research community will generate a rich database of genomic variation including complete populations of bean landraces and improved materials composed of hundreds of accessions. The molecular tools derived from this resource will help breeders to reconstruct the genomic footprint of the development of improved materials, provided that the pedigrees of improved lines are available, and will open alternatives to generate high‐precision genomic tools to guide crosses and to track desired haplotypes.

### Supplemental Information

**Supplemental Table S1.** List of samples sequenced and analyzed in this study.

**Supplemental Table S2**. Pipeline parameters and results. Sample‐specific parameters used to run the NGSEP pipeline for the Illumina WGS data analyzed in this study and the results of the read alignment and variant discovery and genotyping process.

**Supplemental Table S3.** List of inter–gene pool introgressions. Report of inter–gene pool introgressions including genomic locations, background population, population of the introgressed haplotype, number of SNPs genotyped within the region, and scores for the background and introgression populations.

**Supplemental Table S4.** List of interspecific introgressions. Report of interspecific introgressions including genomic locations, background species, species of the introgressed haplotype, number of SNPs genotyped within the region, and scores for *P. acutifolius*, *P. coccineus*, and *P. vulgaris* haplotypes.

**Supplemental Table S5.** List of markers evaluated in this study.

**Supplemental Fig. S1.** Percentage of base pair calls that were different from the reference genome. The percentage of base pair calls that were different from the reference genome serves as a function of the read position for representative samples sequenced at different times. Whereas the average percentage is indicative of the genetic distance between the sample and the reference genome, increases at the 5’ end or at the 3’ end of the reads are related to sequencing.

**Supplemental Fig. S2.** Single nucleotide polymorphism genotyping statistics for SNPs within *P. vulgaris*. Genotype calls are discriminated as homozygous for an alternative (nonreference) allele (blue), heterozygous or heterogeneous (red), and homozygous for the reference allele (gray). The percentage of nonreference genotype calls is displayed as a green line.

**Supplemental Fig. S3.** Intersection of CNVs with unique regions. Intersection of regions with predicted CNVs, discriminating between deletions and duplications, for the accessions sequenced in this study with the 228 Mbp of nonrepetitive regions in the reference genome. The values for the accessions BAT 93 and G19833, both having more than 30× average RD are 21.7 and 1.1 Mbp, respectively.

**Supplemental Fig. S4.** Percentage of missing data in CNV predictions of copy number. Percentage of CNVs that could not be reliably genotyped (A) for each accession, and (B) as a function of the average read depth for the CNVs in nonrepetitive regions (Filter 1).

**Supplemental Fig. S5.** Dendrogram based on copy numbers for high‐quality biallelic large deletions identified via read depth analysis.

**Supplemental Fig. S6.** Single nucleotide polymorphism density for segregating SNPs. Number of SNPs in 1‐Mbp windows across the reference genome for SNPs that segregated between wild pools. These are the SNPs used to predict inter–gene pool introgressions. Pericentromeric regions are shown as a red line.

**Supplemental Script S1.** Read alignment for *P. vulgaris* samples. This script was used to align reads from *P. vulgaris* samples to the reference genome and provided sample‐specific distributions of quality, coverage, and insert length. The parameters are sample ID, minimum insert length, and maximum insert length.

**Supplemental Script S2.** Read alignment for *P. acutifolius* and *P. coccineus*. This script was used to align reads from *P. coccineus* or *P. acutifolius* samples to the reference genome and provided sample‐specific distributions of quality, coverage, and insert length. The parameters are sample ID, minimum insert length, and maximum insert length.

**Supplemental Script S3.** Tandem repeats. This script was used to run the Tandem Repeats Finder to identify tandem repeats in the reference genome.

**Supplemental Script S4.** Find variants with CNV analysis. This script was used to run the FindVariants command of NGSEP to discover SNPs, small indels, and structural variants against the reference genome for individual *P. vulgaris* accessions sequenced in this study, plus the accessions G19833 and BAT 93. The parameters are sample ID, bp to ignore in the 5’ end, and bp to ignore in the 3’ end.

**Supplemental Script S5.** Find variants without CNV analysis. This script was used to run the FindVariants command of NGSEP to discover SNPs and small indels against the reference genome for individual inbred samples. This script was used to discover variants in the individual *P. vulgaris* accessions published by Song et al. (2015) and on the accessions G35346 and G40001 in the species *P. coccineus* and *P. acutifolius*. The parameters are sample ID, bp to ignore in the 5’ end, and bp to ignore in the 3’ end.

**Supplemental Script S6.** Find variants in pools. This script was used to run the FindVariants command of NGSEP to discover SNPs and small indels against the reference genome on samples including pools of accessions. This script was used to discover variants from the reads obtained from the pools published by Schmutz et al. (2014). The parameters are sample ID, bp to ignore in the 5’ end, and bp to ignore in the 3’ end.

**Supplemental Script S7.** Genotype variants with CNV analysis. This script was used to run the FindVariants command of NGSEP to genotype a dataset of known SNPs, small indels, and CNVs. The catalog of known SNPs and small indels was assembled with the “MergeVariants” command of NGSEP to merge the per‐sample VCF files produced at the discovery step (Supplemental Script S4, Supplemental Script S5 and Supplemental Script S6). The catalog of known CNVs was assembled for this work following the heuristic procedure described in the Materials and Methods section. This script was used to genotype the individual *P. vulgaris* accessions sequenced in this study, plus the accessions G19833 and BAT 93. The parameters are sample ID, bp to ignore in the 5’ end, and bp to ignore in the 3’ end.

**Supplemental Script S8.** Genotype variants without CNV analysis. This script was used to run the FindVariants command of NGSEP to genotype a dataset of known SNPs and small indels on individual samples. This script was used to discover variants in the individual *P. vulgaris* accessions published by Song et al. (2015) and on the accessions G35346 and G40001 of the species *P. coccineus* and *P. acutifolius*. The catalog of known SNPs and small indels was assembled with the “MergeVariants” command of NGSEP to merge the per‐sample VCF files produced at the discovery step (Supplemental Script S4, Supplemental Script S5 and Supplemental Script S6). The parameters are sample ID, bp to ignore in the 5’ end, and bp to ignore in the 3’ end.

**Supplemental Script S9.** Genotype variants in pools. This script was used to run the FindVariants command of NGSEP to genotype a dataset of known SNPs and small indels on samples including pools of accessions. This script was used to genotype variants from the reads obtained from the pools published by Schmutz et al. (2014). The catalog of known SNPs and small indels was assembled with the “MergeVariants” command of NGSEP to merge the per‐sample VCF files produced at the discovery step (Supplemental Script S4, Supplemental Script S5 and Supplemental Script S6). The parameters are sample ID, bp to ignore in the 5’ end, and bp to ignore in the 3’ end.

### Conflict of Interest Disclosure

The authors declare that there are no conflicts of interest related to this manuscript.

## Supporting information

Supplemental Information
Supplemental File 1

Supplemental Information
Supplemental File 2

Supplemental Information
Supplemental File 3

Supplemental Information
Supplemental File 4

Supplemental Information
Supplemental File 5

Supplemental Information
Supplemental File 6
