# Supplementary material for: Resequencing of Common Bean Identifies Regions of Inter–Gene Pool Introgression and Provides Comprehensive Resources for Molecular Breeding
Source: Plant Genome. 2018 Jul 1;11(2):170068. doi: 10.3835/plantgenome2017.08.0068 (PMC12810085; doi:10.3835/plantgenome2017.08.0068)
Supplement: Supplementary file 1 [file TPG2-11-170068-s001.pdf]

Supplemental Tables, Figures and Scripts for:

Re-sequencing of common bean identifies regions of inter-gene pool introgression and provides comprehensive resources for molecular breeding

Juan David Lobaton[1,†,#], Tamara Miller[2,†], Juanita Gil[1], Daniel Ariza[1], Juan Fernando de la Hoz[1], Alvaro Soler[1,+], Steve Beebe[1], Jorge Duitama[1,3,*], Paul Gepts[2,‡], Bodo Raatz[1,‡]

1 Agrobiodiversity Research Area, International Center for Tropical Agriculture (CIAT), Cali, Colombia

2 Department of Plant Sciences, University of California, Davis, USA

3 Systems and Computing Engineering Department, Universidad de los Andes, Bogotá, Colombia

# Current address: School of Environmental and Rural Science, University of New England, Armidale, Australia

+ Current address: SDA-ARS, Grain Legume Genetics and Physiology Research Unit, Prosser, WA, USA

*** Corresponding author**

E-mail : ja.duitama@uniandes.edu.co

† These authors contributed equally to this work.

‡ PG and BR are joint last authors

Supplemental Table S1. List of samples sequenced and analyzed in this study. (Separate excel file).

Supplemental Table S2. Pipeline parameters and results. Sample-specific parameters used to run the NGSEP pipeline for the Illumina WGS data analyzed in this study and results of the read alignment and variant discovery and genotyping process. (Separate excel file).

Supplemental Table S3. List of inter-gene pool introgressions. Report of inter-gene pool introgressions including genomic locations, background population, population of the introgressed haplotype, number of SNPs genotyped within the region and scores for the background, and introgression populations. (Separate excel file).

Supplemental Table S4. List of interspecific introgressions. Report of interspecific introgressions including genomic locations, background species, species of the introgressed haplotype, number of SNPs genotyped within the region and scores for *P. Acutifolius*, *P. Coccineus* and *P. Vulgaris* haplotypes. (Separate excel file)

Supplemental Table 5. List of markers evaluated in this study (Separate excel file).

Supplemental Fig. S1. Percentage of base pair calls different than the reference genome. The percentage of base pair calls different than the reference genome serves as a function of the read position for representative samples sequenced at different times. Whereas the average percentage is indicative of the genetic distance between the sample and the reference genome, increases at the 5' end or at the 3' end of the reads are related to sequencing.

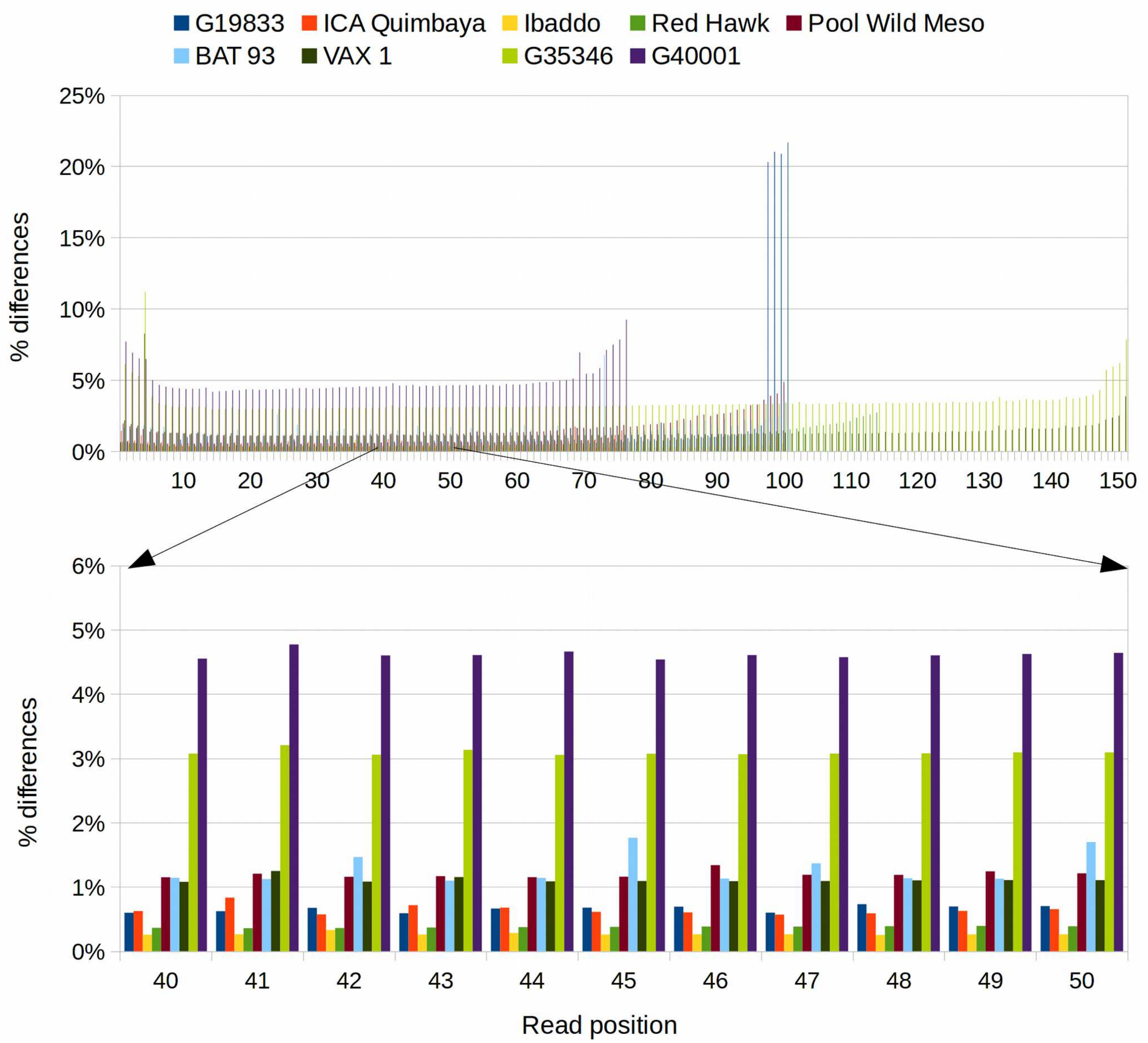

Supplemental Fig. S2. SNP genotyping statistics for SNPs within *P. vulgaris*. Genotype calls are discriminated as homozygous for an alternative (non-reference) allele (blue), heterozygous / heterogeneous (red), and homozygous for the reference allele (gray). The percentage of non-reference genotype calls is displayed as a green line.

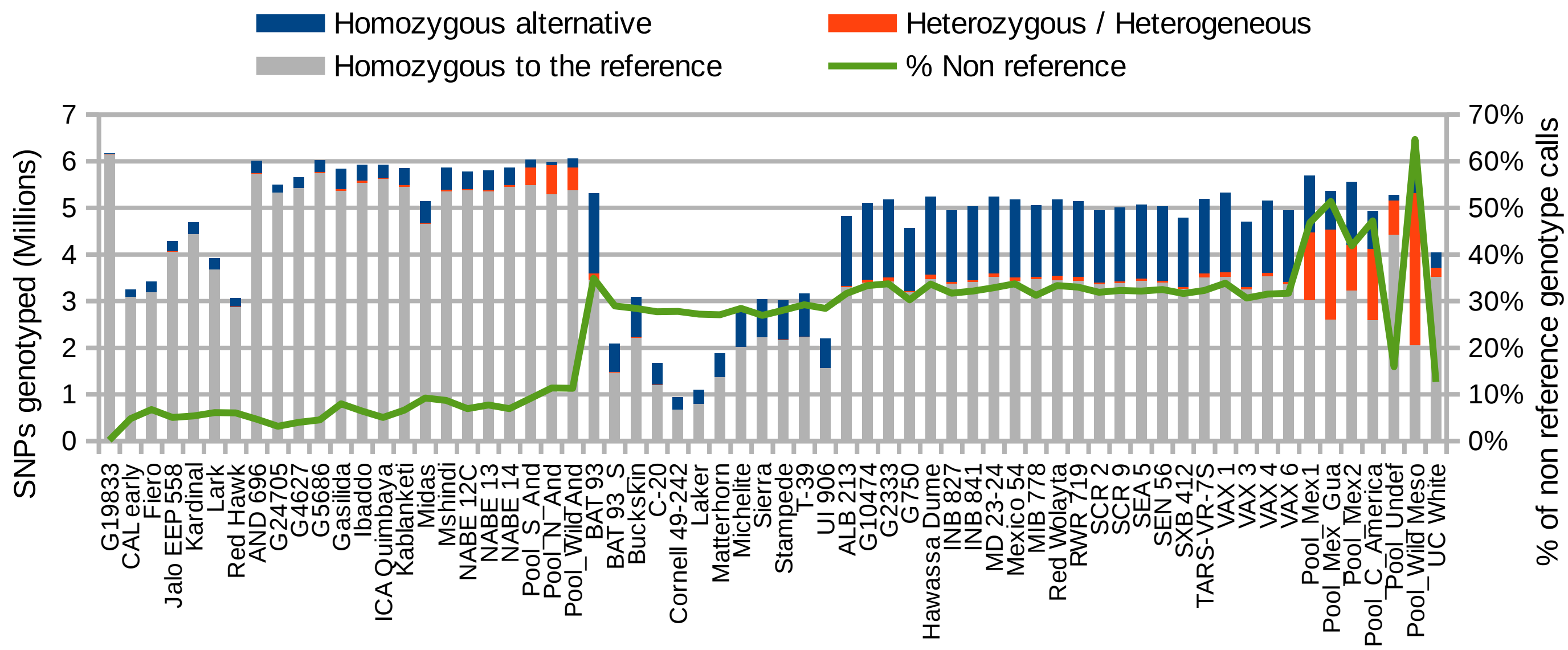

Supplemental Fig. S3. Intersection of CNVs with unique regions. Intersection of regions with predicted CNVs, discriminated between deletions and duplications, for the accessions sequenced in this study with the 228 Mbp of non-repetitive regions in the reference genome. The values for the accessions BAT 93 and G19833, both having more than 30x average RD are 21.7Mbp and 1.1Mbp respectively

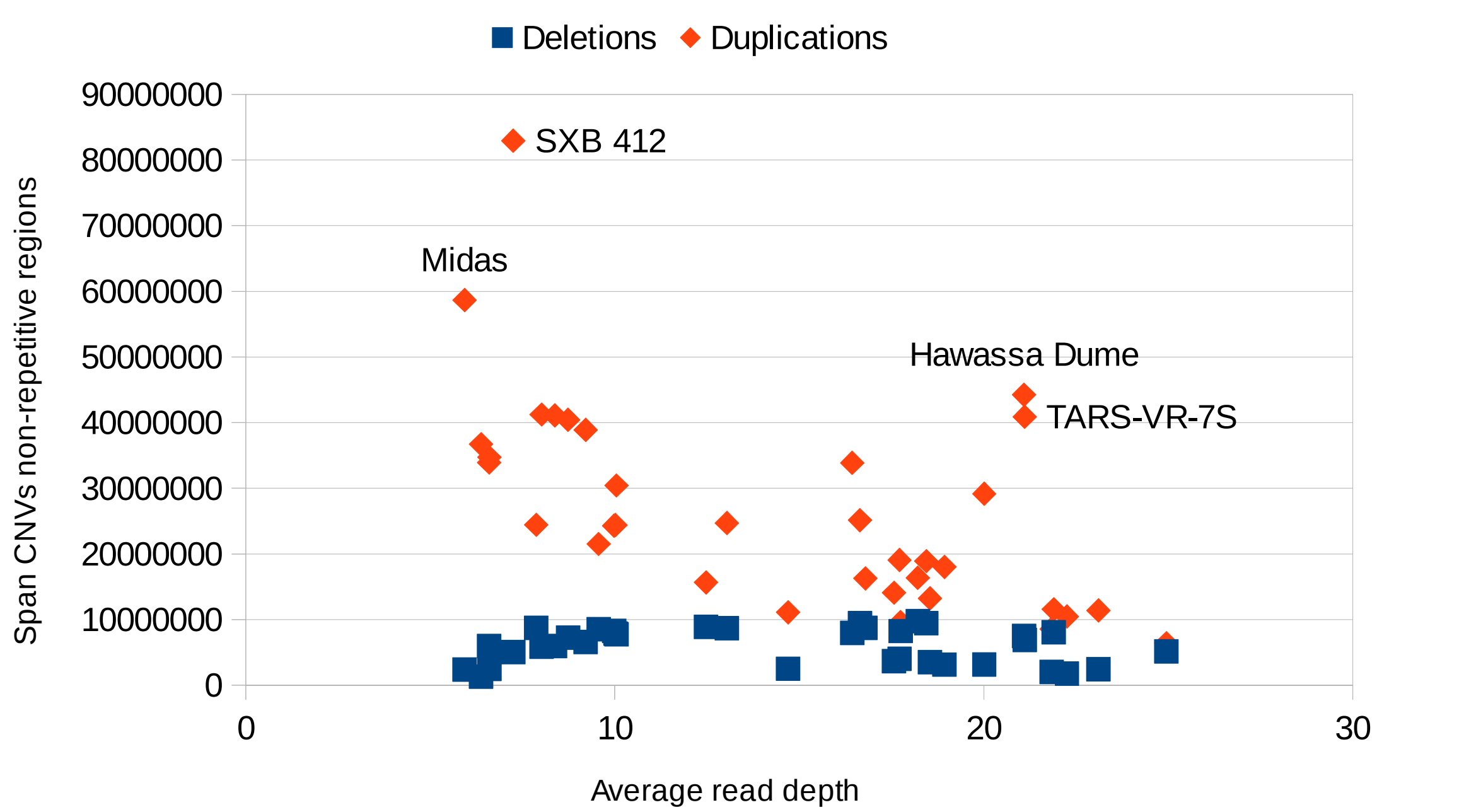

Supplemental Fig. S4. Percentage of missing data in CNV predictions of copy number. Percentage of CNVs that could not be reliably genotyped (A) for each accession, and (B) as a function of the average read depth for the CNVs in non-repetitive regions (Filter 1)

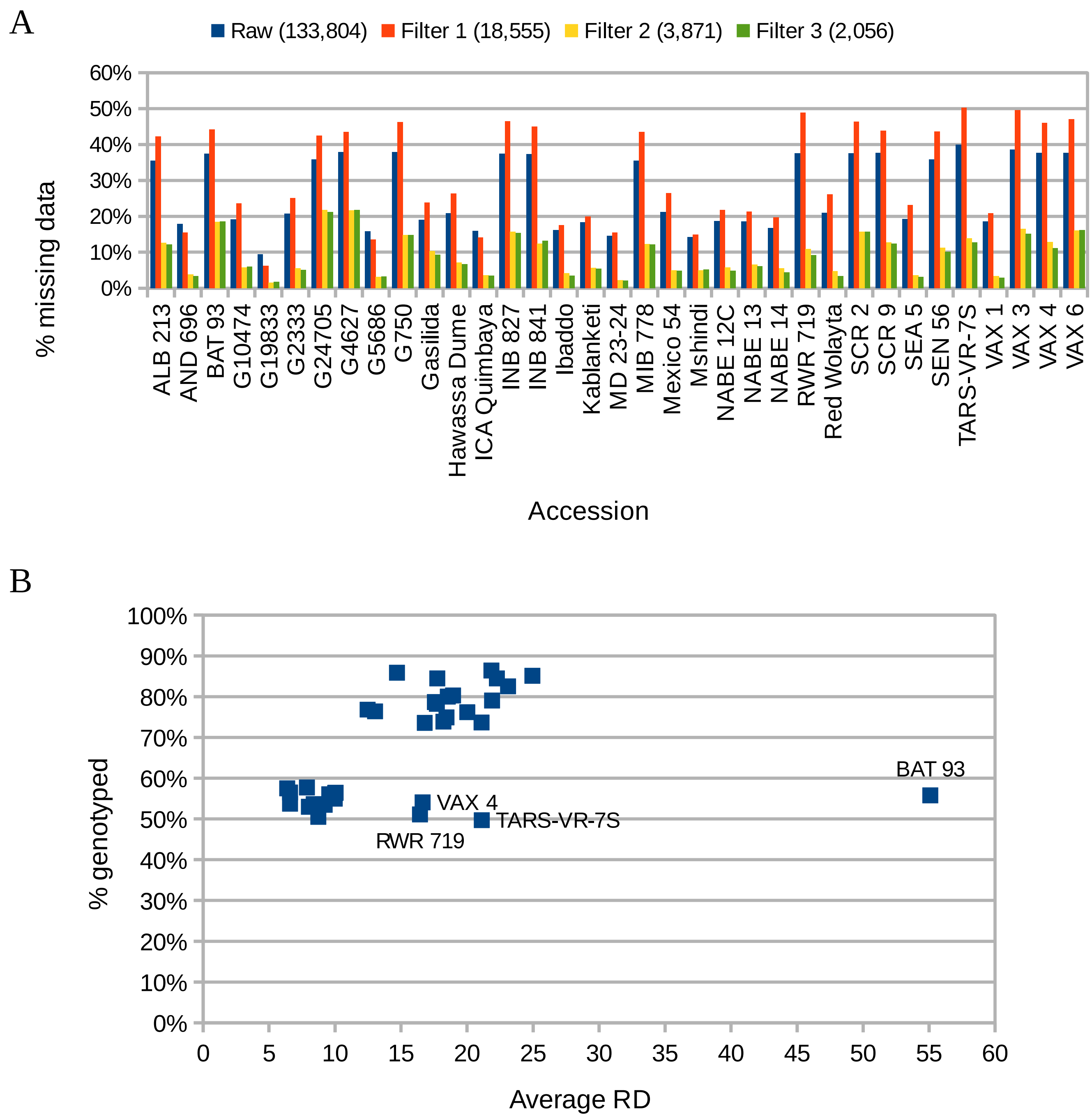

Supplemental Fig. S5. Dendogram based on copy number for high quality biallelic large deletions identified with read depth analysis

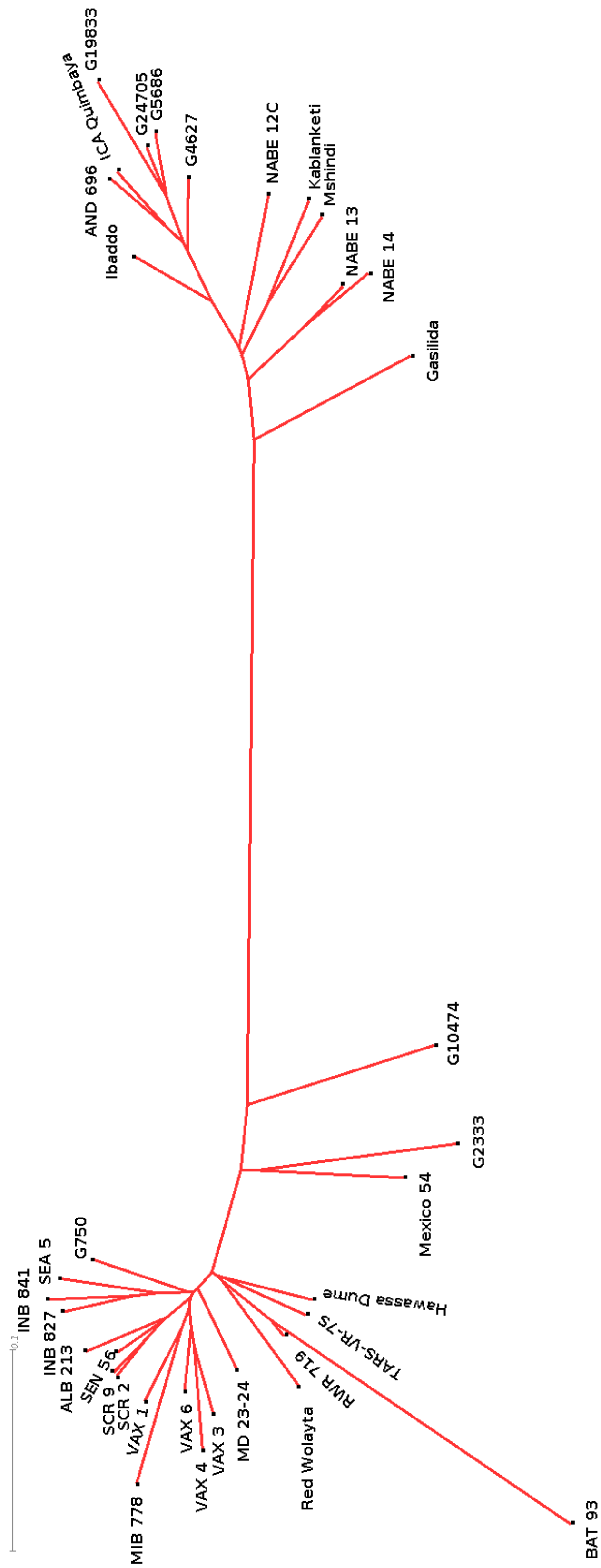

Supplemental Fig. S6. SNP density for segregating SNPs. Number of SNPs in 1 Mbp windows across the reference genome for SNPs that segregate between wild pools. These are the SNPs used to predict inter-gene pool introgressions. Pericentromeric regions are shown as a red line

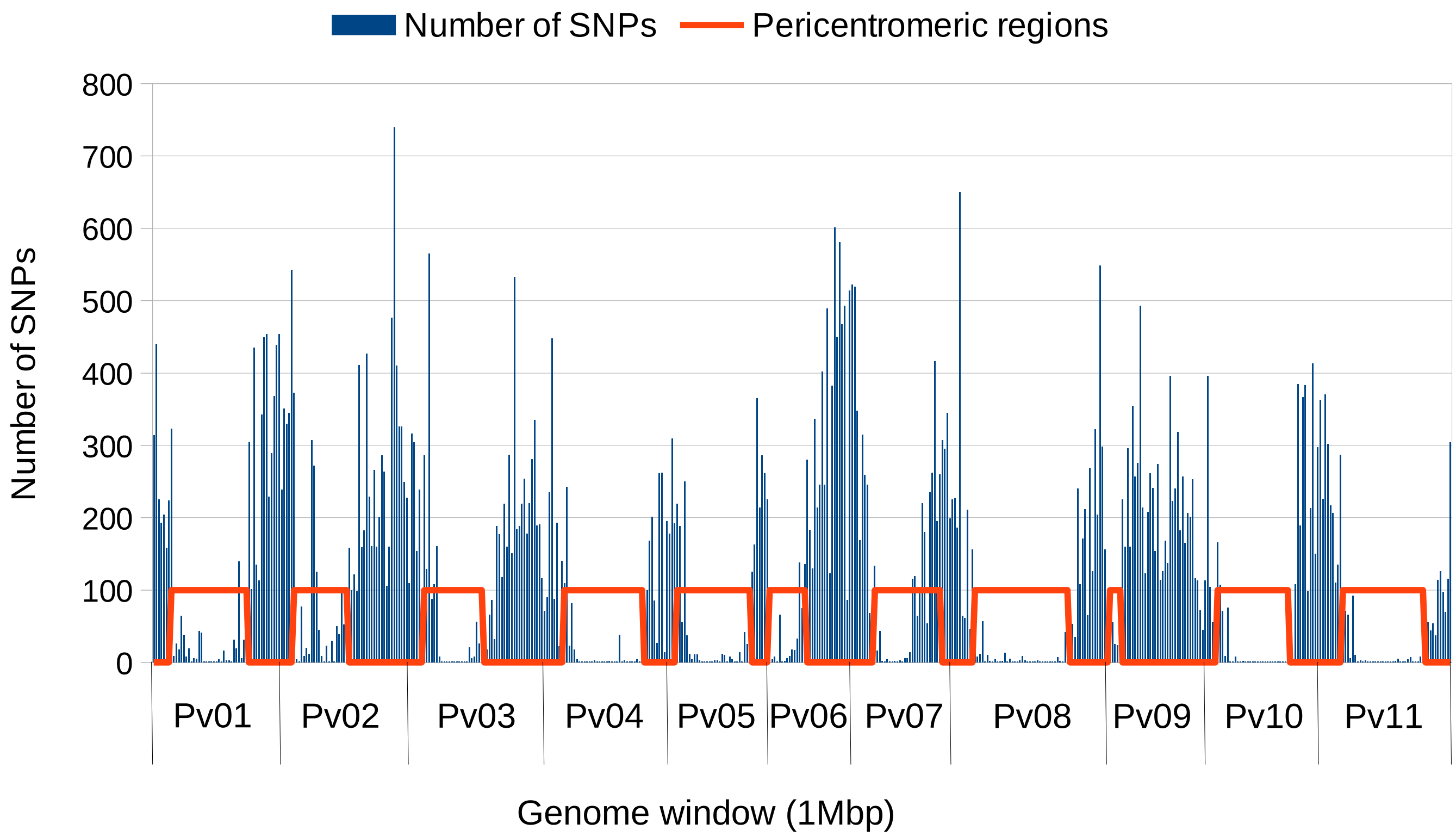

Supplemental script S1. Read alignment for *P. vulgaris* samples. Script used to align reads from *P. Vulgaris* samples to the reference genome and provide sample specific distributions of quality, coverage and insert length. The parameters are sample id, minimum insert length and maximum insert length

```
p=$1;
i=$2;
x=$3;
s=SM:${p};
f1=${p}_1.fastq.gz;
f2=${p}_2.fastq.gz;

REFERENCE=Pvulgaris_442_v2.0.fa;
BOWTIE2=bowtie2;
SAMTOOLS=samtools;
PICARD=picard.jar;
NGSEP=NGSEPcore_3.0.2.jar;

$BOWTIE2 --rg-id ${p} --rg ${s} --rg PL:ILLUMINA  -I ${i} -X ${x} -p 2 -k 3 -t -x $REFERENCE -1 ${f1}
-2 ${f2} 2> ${p}_bowtie2.log | ${SAMTOOLS} view -bhS - > ${p}_bowtie2.bam
mkdir ${p}_tmpdir;
java -Xmx8g -jar ${PICARD} SortSam MAX_RECORDS_IN_RAM=1000000 SO=coordinate CREATE_INDEX=true TMP_DIR=$
{p}_tmpdir I=${p}_bowtie2.bam O=${p}_bowtie2_sorted.bam >& ${p}_bowtie2_sort.log;
rm -rf ${p}_tmpdir;
rm -f ${p}_bowtie2.bam;
java -Xmx3g -jar ${NGSEP} QualStats ${REFERENCE} ${p}_bowtie2_sorted.bam >& ${p}_bowtie2_readpos.stats;
java -Xmx3g -jar ${NGSEP} CoverageStats ${p}_bowtie2_sorted.bam ${p}_bowtie2_coverage.stats >& $
{p}_bowtie2_coverage.log;
${SAMTOOLS} view -F 268 ${p}_bowtie2_sorted.bam | awk '{l=$9;if(l>=0)
{i=sprintf("%d",l/25)+1;if(i<100)a[i]++;else aM++}}END{for(i=1;i<100;i++)print (i-1)*25,a[i];print
"More",aM}' >& ${p}_bowtie2_insertLength.stats;
```

Supplemental script S2. Read alignment for *P. acutifolius* and *P. coccineus*. Script used to align reads from *P. Coccineus* or *P. Acutifolius* samples to the reference genome and provide sample specific distributions of quality, coverage and insert length. The parameters are sample id, minimum insert length and maximum insert length.

```
p=$1;
i=$2;
x=$3;
s=SM:${p};
f1=${p}_1.fastq.gz;
f2=${p}_2.fastq.gz;

REFERENCE=Pvulgaris_442_v2.0.fa
BOWTIE2=bowtie2
SAMTOOLS=samtools
PICARD=picard.jar
NGSEP=NGSEPcore_3.0.2.jar

$BOWTIE2 --rg-id ${p} --rg ${s} --rg PL:ILLUMINA -D 25 -R 4 -N 1 -L 17 -i S,1,0.3 --rdg 4,2 --rfg 4,2
--n-ceil L,0,0.33 --score-min L,-1,-1 -I ${i} -X ${x} --np 0 -k 3 -t -p 2 -x $REFERENCE -1 ${f1} -2 $
{f2} 2> ${p}_bowtie2.log | ${SAMTOOLS} view -bhS - > ${p}_bowtie2.bam
mkdir ${p}_tmpdir
java -Xmx10g -jar ${PICARD} SortSam MAX_RECORDS_IN_RAM=1000000 SO=coordinate CREATE_INDEX=true
TMP_DIR=${p}_tmpdir I=${p}_bowtie2.bam O=${p}_bowtie2_sorted.bam >& ${p}_bowtie2_sort.log
rm -rf ${p}_tmpdir
rm -f ${p}_bowtie2.bam;
java -Xmx3g -jar ${NGSEP} QualStats ${REFERENCE} ${p}_bowtie2_sorted.bam >& ${p}_bowtie2_readpos.stats
java -Xmx3g -jar ${NGSEP} CoverageStats ${p}_bowtie2_sorted.bam ${p}_bowtie2_coverage.stats >& $
{p}_bowtie2_coverage.log
${SAMTOOLS} view -F 268 ${p}_bowtie2_sorted.bam | awk '{l=$9;if(l>=0)
{i=sprintf("%d",l/25)+1;if(i<100)a[i]++;else aM++}}END{for(i=1;i<100;i++)print (i-1)*25,a[i];print
"More",aM}' >& ${p}_bowtie2_insertLength.stats
```

Supplemental script S3. Tandem repeats. Script used to run Tandem Repeats Finder to identify tandem repeats in the reference genome.

```
trf409.linux64 Pvulgaris_442_v2.0.fa 2 7 7 80 10 20 50 -h -ngs | awk '{if(substr($0,1,1)=="@")
c=substr($0,2);else print c,$0}' > Pvulgaris_442_v2.0.fa.2.7.7.80.10.20.50.ngs

# parameters explained:
# match=2, missmatch=-7, indel=-7
# ProbabilityMatch=80 ProbabilityIndel=10 (empirical suggested values)
# minAlignmentScore=20
# maxSizeOfTandemPattern=50

awk '{if( $4==1 || $4==2 || $5>=3 || $9>40 ) print $0}' Pvulgaris_442_v2.0.fa.2.7.7.80.10.20.50.ngs >
Pvulgaris_v2_strs.list

# filter explained:
# $4 -> length of the tandem pattern (1 for AAAA, CCCC, etc. 2 for ATATAT, GCGCGC, CTCTCT, etc)
# $5 -> number of tandem repeats (2.5 would be 'ATATA' for pattern AT)
# $9 -> alignment score (match=2,missmatch=-7,indel=-7)
```

Supplemental script S4. Find variants with CNV analysis. Script used to run the FindVariants command of NGSEP to discover SNPs, small indels and structural variants against the reference genome for individual *P. Vulgaris* accessions sequenced in this study, plus the acccesions G19833 and BAT 93. The parameters are sample id, bp to ignore in the 5' end and bp to ignore in the 3' end

```
p=$1;
f=$2;
t=$3;

REFERENCE=Pvulgaris_442_v2.0.fa
NGSEP=NGSEPcore_3.0.2.jar
STRS=Pvulgaris_v2_strs.list

java -Xmx4g -jar ${NGSEP} FindVariants -noRep -ignore5 ${f} -ignore3 ${t} -maxBaseQS 30 -minQuality 40
-h 0.0001 -knownSTRs ${STRS} -maxAlnsPerStartPos 2 -sampleId ${p} ${REFERENCE} ${p}_bowtie2_sorted.bam
${p}_bowtie2_NGSEP >& ${p}_bowtie2_NGSEP.log;
```

Supplemental script S5. Find variants without CNV analysis. Script used to run the FindVariants command of NGSEP to discover only SNPs and small indels against the reference genome for individual inbred samples. This script was used to discover variants in the individual *P. Vulgaris* accessions published by Song et al. 2015 and on the accessions G35346 and G40001 of the species *P. Coccineus* and *P. Acutifolius*. The parameters are sample id, bp to ignore in the 5' end and bp to ignore in the 3' end

```
p=$1;
f=$2;
t=$3;

REFERENCE=Pvulgaris_442_v2.0.fa
NGSEP=NGSEPcore_3.0.2.jar
STRS=Pvulgaris_v2_strs.list

java -Xmx4g -jar ${NGSEP} FindVariants -noRep -noRD -noRP -ignore5 ${f} -ignore3 ${t} -maxBaseQS 30
-minQuality 40 -h 0.0001 -knownSTRs ${STRS} -maxAlnsPerStartPos 2 -sampleId ${p} ${REFERENCE} $
{p}_bowtie2_sorted.bam ${p}_bowtie2_NGSEP >& ${p}_bowtie2_NGSEP.log;
```

Supplemental script S6. Find variants in pools. Script used to run the FindVariants command of NGSEP to discover only SNPs and small indels against the reference genome on samples including pools of accessions. This script was used to discover variants from the reads obtained from the pools published by Schmutz et al. 2014. The parameters are sample id, bp to ignore in the 5' end and bp to ignore in the 3' end

```
p=$1;
f=$2;
t=$3;

REFERENCE=Pvulgaris_442_v2.0.fa
NGSEP=NGSEPcore_3.0.2.jar
STRS=Pvulgaris_v2_strs.list

java -Xmx4g -jar ${NGSEP} FindVariants -noRep -noRD -noRP -ignore5 ${f} -ignore3 ${t} -maxBaseQS 30
-minQuality 40 -knownSTRs ${STRS} -maxAlnsPerStartPos 2 -sampleId ${p} ${REFERENCE} $
{p}_bowtie2_sorted.bam ${p}_bowtie2_NGSEP >& ${p}_bowtie2_NGSEP.log
```

Supplemental script S7. Genotype variants with CNV analysis. Script used to run the FindVariants command of NGSEP to genotype a dataset of known SNPs, small indels and CNVs. The catalog of known SNPs and small indels is assembled using the "MergeVariants" command of NGSEP, to merge the per-sample VCF files produced at the discovery step (S4-S6 Scripts). The catalog of known CNVs was assembled for this work following the heuristic procedure described in the Methods section "Analysis of copy number variation". This script was used to genotype the individual *P. Vulgaris* accessions sequenced in this study, plus the acccesions G19833 and BAT 93. The parameters are sample id, bp to ignore in the 5' end and bp to ignore in the 3' end

```
p=$1;
f=$2;
t=$3;

REFERENCE=Pvulgaris_442_v2.0.fa
NGSEP=NGSEPcore_3.0.2.jar
SVS=knownSVs.gff
VARS=Variants_WGS_tpg_noGenotype.vcf

java -Xmx4g -jar ${NGSEP} FindVariants -noRep -noRP -noNewCNV -minSVQuality 0 -ignore5 ${f} -ignore3 $
{t} -maxBaseQS 30 -minQuality 0 -h 0.0001 -knownSVs ${SVS} -knownVariants ${VARS} -maxAlnsPerStartPos 2
-sampleId ${p} ${REFERENCE} ${p}_bowtie2_sorted.bam ${p}_bowtie2_NGSEP_gt >& ${p}_bowtie2_NGSEP_gt.log
```

Supplemental script S8. Genotype variants without CNV analysis. Script used to run the FindVariants command of NGSEP to genotype only a dataset of known SNPs and small indels on individual samples. This script was used to discover variants in the individual *P. Vulgaris* accessions published by Song et al. 2015 and on the accessions G35346 and G40001 of the species *P. Coccineus* and *P. Acutifolius*. The catalog of known SNPs and small indels is assembled using the "MergeVariants" command of NGSEP, to merge the per-sample VCF files produced at the discovery step (S4-S6 Scripts). The parameters are sample id, bp to ignore in the 5' end and bp to ignore in the 3' end

```
p=$1;
f=$2;
t=$3;

REFERENCE=Pvulgaris_442_v2.0.fa
NGSEP=NGSEPcore_3.0.2.jar
VARS=Variants_WGS_tpg_noGenotype.vcf

java -Xmx4g -jar ${NGSEP} FindVariants -noRep -noRD -noRP -ignore5 ${f} -ignore3 ${t} -maxBaseQS 30
-minQuality 0 -h 0.0001 -knownVariants ${VARS} -maxAlnsPerStartPos 2 -sampleId ${p} ${REFERENCE} $
{p}_bowtie2_sorted.bam ${p}_bowtie2_NGSEP_gt >& ${p}_bowtie2_NGSEP_gt.log;
```

Supplemental script S9. Genotype variants in pools. Script used to run the FindVariants command of NGSEP to genotype only a dataset of known SNPs and small indels on samples including pools of accessions. This script was used to genotype variants from the reads obtained from the pools published by Schmutz et al. 2014. The catalog of known SNPs and small indels is assembled using the “MergeVariants” command of NGSEP, to merge the per-sample VCF files produced at the discovery step (S4-S6 Scripts). The parameters are sample id, bp to ignore in the 5’ end and bp to ignore in the 3’ end

```
p=$1;
f=$2;
t=$3;

REFERENCE=Pvulgaris_442_v2.0.fa
NGSEP=NGSEPcore_3.0.2.jar
VARS=Variants_WGS_tpg_noGenotype.vcf

java -Xmx4g -jar ${NGSEP} FindVariants -noRep -noRD -noRP -ignore5 ${f} -ignore3 ${t} -maxBaseQS 30
-minQuality 0 -knownVariants ${VARS} -maxAlnsPerStartPos 2 -sampleId ${p} ${REFERENCE} $
{p}_bowtie2_sorted.bam ${p}_bowtie2_NGSEP_gt >& ${p}_bowtie2_NGSEP_gt.log
```
